# Supplementary material for: Communicating biopsy results from breast screening assessment: current practice in English breast screening centres and staff perspectives of telephoning results
Source: BMJ Open. 2019 Nov 7;9(11):e028683. doi: 10.1136/bmjopen-2018-028683 (PMC6858119; doi:10.1136/bmjopen-2018-028683)
Supplement: Supplementary data [file bmjopen-2018-028683supp001.pdf]

The communication of benign biopsy results in the NHS Breast Screening Programme – Online centre survey

CENTRE CHARACTERISTICS

1. Please state the official name of your breast screening centre:

   ____

2. What age group of women does your centre screen most frequently?
   (this information for your centre can be found in the KC62)

   ☐ <=44
   ☐ 45-49
   ☐ 50-52
   ☐ 53-54
   ☐ 55-59
   ☐ 60-64
   ☐ 65-69
   ☐ 70
   ☐ 71-74
   ☐ >=75

1st April 2017 – V.3

COMMUNICATION OF RESULTS

3. When delivering benign biopsy results…

   Women are never telephoned with results ☐
   Women are occasionally telephoned with results ☐
   Women are routinely telephoned with results ☐

   Please add any additional comments below:

   ____
   ____
   ____

4. If women ***are*** telephoned with benign results, who delivers these results? (Tick all that apply)

   Clinical nurse specialist ☐
   Radiologist ☐
   Radiographer ☐
   Breast care surgeon ☐
   Administrative staff ☐
   Other (please specify): ☐

   ____

1st April 2017 – V.3

5. When delivering a cancer result…

   Women are never telephoned with results ☐
   Women are occasionally telephoned with results ☐
   Women are routinely telephoned with results ☐

   Please add any additional comments below:

   ____
   ____
   ____

6. If women ***are*** telephoned with cancer results, who delivers these results? (Tick all that apply)

   Clinical nurse specialist ☐
   Radiologist ☐
   Radiographer ☐
   Breast care surgeon ☐
   Administrative staff ☐
   Other (please specify): ☐

   ____

1st April 2017 – V.3

7. From assessment to delivering a result, how long does this process take (on average)?
   1 day ☐
   2 days ☐
   3 days ☐
   4 days ☐
   5 days ☐
   6 days ☐
   7 days ☐
   8 days ☐
   9 days ☐
   10 days ☐
   11 days ☐
   12 days ☐
   More than 12 days ☐

8. We are currently recruiting breast screening centres to take part in our main study, which will involve surveying women attending breast screening, who go to receive a benign result. The surveys will be assessing patient anxiety and understanding of results, examining whether the method of communication used (telephone or in-person) to deliver benign results influences these patient outcomes. We are looking for two centres to participate, to allocate women to receive benign results via either telephone or in-person. We are currently in the process of finalising the design of methodology and submitting for ethical approval.

   Would you be interested in involvement in this research?

   Yes ☐
   No ☐

If you select yes, you may be contacted soon with further details of the main study.

9. Would your centre like to know the results from this survey? (If yes, please provide an email address for a summary sheet to be sent upon completion of the research).

No ☐
Yes ☐

Email address: ____________________________________

1st April 2017 – V.3

Thank you for taking the time to participate in this survey.

If you have any questions, feel free to contact the lead researcher:

Lead researcher: Sian Williamson

Email: s.williamson@warwick.ac.uk

Telephone: 07891319509

1st April 2017 – V.3
