# Supplementary material for: Communicating biopsy results from breast screening assessment: current practice in English breast screening centres and staff perspectives of telephoning results
Source: BMJ Open. 2019 Nov 7;9(11):e028683. doi: 10.1136/bmjopen-2018-028683 (PMC6858119; doi:10.1136/bmjopen-2018-028683)
Supplement: Supplementary data [file bmjopen-2018-028683supp002.pdf]

## Appendix 2 – Qualitative content analysis data in tabular form

**When delivering benign (non-cancer) biopsy results are women never telephoned with results, occasionally telephoned with results or routinely telephoned with results?**

| Content | Comment |
|---|---|
| Centres schedule an in-person appointment for the woman to receive results but then telephone ahead of this appointment if results are benign (n=7) | *"Women have a scheduled face to face appointment for results but if it's benign we ring them." (Centre ID 02)*<br><br>*"Our aim is to call all the benign results and offer to cancel the booked appointment." (Centre ID 50)*<br><br>*"All women are given a results appointment during assessment clinic. Following MDT, all those with benign biopsy results are contacted by telephone. If contact is made, the result is discussed and the results appointment cancelled." (Centre ID 38)*<br><br>*"Women are seen in the assessment clinic and are asked by the BCN for permission to ring if need be to alter appointments etc. but we do not inform them that we will with the results in case it is more appropriate for them to come to clinic to receive the results, all patients leave clinic with an appointment" (Centre ID 34)*<br><br>*"All patients are given an appointment to attend for results we do telephone with results." (Centre ID 45)*<br><br>*"Women always have an appointment booked [but are telephoned if benign]" – (Centre ID 28)*<br><br>*"Appointments are made for all results but benign results are sometimes delivered over the phone, where women have been expecting a benign result." – (Centre ID 09)* |
| In-person communication is routine practice for delivering benign results (n=1) | *"We normally see women face to face." (Centre ID 25)* |
| Centres who telephone with results offer the woman the option to still attend in-person for further support (n=5) | *"All patients are given an appointment to attend for results we do telephone with results but patients are still able to attend, and some do." (Centre ID 45)*<br><br>*"After the MDT. Patients are telephoned with benign results by a qualified Breast Care Nurse. They are then offered an OPA with a consultant surgeon if they have concerns." (Centre ID 31)*<br><br>*"Our aim is to call all the benign results and offer to cancel the booked appointment, but we always stress they can come in anyway –no-one ever does!" (Centre ID 50)*<br><br>*"Women always have an appointment booked and are still offered to come to see a clinician if they still wish." (Centre ID 28)*<br><br>*"All women are given a results appointment during assessment clinic. Following MDT, all those with benign biopsy results are contacted by telephone. If contact is made, the result is discussed and the results appointment cancelled unless the woman specifically wants to attend for further information/support." (Centre ID 38)* |
| Centres only telephone benign results in rare and exceptional circumstances, where the woman cannot return in-person (n=5) | *"Only in exceptional circumstances." (Centre ID 59)*<br><br>*"This is not done routinely and very rarely occurs." (Centre ID 78)*<br><br>*"In exceptional circumstances." (Centre ID 46)*<br><br>*"Majority return for results, only rung if they have issues returning." (Centre ID 24)*<br><br>*"We normally see ladies face to face but will phone them if they are unable to attend their appointment." (Centre ID 25)* |
| Centres offer women the choice | *"We will always offer them an appointment to come in, but the BCNs will ask if they want a telephone call at the time of assessment." (Centre ID 63)* |

| | |
|---|---|
| of how they would prefer to receive their result (n=5) | *"Women are asked at assessment if they would like a telephone call or they can come back for results if they do not wished to be telephoned." (Centre ID 29)*<br><br>*"Women are given a choice about how they receive their results when the imaging suggests a benign process." (Centre ID 42)*<br><br>*"Women call in to us for a result as prearranged with specialist nurses. So they are where they want to be to get the result. The BCN will discuss with them beforehand the different outcomes that are possible. It is always explained that the result maybe different than expected and how they would feel about that." (Centre ID 01)*<br><br>*Prior discussion with the patient – (Centre ID 46)* |
| Centres will telephone women if the patient makes this request (n=1) | *"This is not routine practice but happens if a patient requests it and the probability of a benign result is very high." (Centre ID 73)* |

**When delivering cancer biopsy results are women never telephoned with results, occasionally telephoned with results or routinely telephoned with results?**

| Content | Comment |
|---|---|
| In-person communication is routine practice for delivering cancer results (n=4) | *"Cancer diagnoses are always communicated face to face." (Centre ID 39)*<br><br>*"[telephone results] This would never be planned." (Centre ID 09)*<br><br>*"All positive results or complicated cases are invited back to be given results by the Breast Surgery Team." (Centre ID 26)*<br><br>*"Routinely they are brought back for a face to face appointment." (Centre ID 02)* |
| Centres only telephone cancer results in rare and exceptional circumstances, where the woman cannot return in-person (n=11) | *"Rarely telephoned with a cancer diagnosis always at the patients request in extenuating circumstances." (Centre ID 45)*<br><br>*"This is a rare occurrence and is only agreed to with the patients prior consent on the understanding they may be receiving a cancer diagnosis." (Centre ID 11)*<br><br>*"Very rare - this would only happen with prior agreement if a woman is to be away for an extended period of time." (Centre ID 38)*<br><br>*"On rare occasions." (Centre ID 05)*<br><br>*"Except on one occasion." (Centre ID 15)*<br><br>*"Cancer results only discussed by telephone in the event that a woman refuses a face to face clinic appointment." (Centre ID 76)*<br><br>*"In exceptional circumstances." (Centre ID 46)*<br><br>*"Only in extreme circumstances and with much counselling beforehand - such as will be on holiday for months. This happens maybe once or twice a year." (Centre ID 50)*<br><br>*"Only very occasionally in exceptional circumstances." (Centre ID 17)*<br><br>*"This is very rare." (Centre ID 18)*<br><br>*"Only very rarely." (Centre ID 08)* |
| Centres offer women the choice of how they would prefer to receive their result (n=2) | *"They are asked if the result was a surprise and was a breast cancer would you still wish to get that news over the phone." (Centre ID 01)*<br><br>*"Women are asked at assessment if they would like a telephone call or they can come back for results if they do not wished to be telephoned." (Centre ID 29)* |
| Centres will telephone women if the patient makes this request (n=5) | *"Patient request only." (Centre ID 33)*<br><br>*"At patient's specific request." (Centre ID 08)*<br><br>*"At patient request." (Centre ID 05)*<br><br>*"Women are only telephoned with a cancer result if they have specifically asked for it that way and signed to give their consent." (Centre ID 02)*<br><br>*"Always at the patients request in extenuating circumstances." (Centre ID 45)* |
| What happens when a benign result is expected, a telephone appointment is arranged but the outcome is an unexpected cancer (n=1) | *"If there is a positive result which was unexpected a Breast Care Nurse rings the woman to advise an appointment is required to discuss the results." (Centre ID 60)* |

| | |
|---|---|
| A negative patient reaction to receiving cancer diagnosis by telephone (n=1) | *"Reacted extremely badly on telephone" (Centre ID 15)* |
