# Supplementary material for: Communicating biopsy results from breast screening assessment: current practice in English breast screening centres and staff perspectives of telephoning results
Source: BMJ Open. 2019 Nov 7;9(11):e028683. doi: 10.1136/bmjopen-2018-028683 (PMC6858119; doi:10.1136/bmjopen-2018-028683)
Supplement: Author's manuscript [file bmjopen-2018-028683.draft_revisions.pdf]

BMJ Open is committed to open peer review. As part of this commitment we make the peer review history of every article we publish publicly available.

When an article is published we post the peer reviewers' comments and the authors' responses online. We also post the versions of the paper that were used during peer review. These are the versions that the peer review comments apply to.

The versions of the paper that follow are the versions that were submitted during the peer review process. They are not the versions of record or the final published versions. They should not be cited or distributed as the published version of this manuscript.

BMJ Open is an open access journal and the full, final, typeset and author-corrected version of record of the manuscript is available on our site with no access controls, subscription charges or pay-per-view fees (http://bmjopen.bmj.com).

If you have any questions on BMJ Open's open peer review process please email info.bmjopen@bmj.com

BMJ Open

Telephone results services in English breast screening centres: a survey of current communication trends and issues

Journal:	*BMJ Open*
Manuscript ID	bmjopen-2018-028683
Article Type:	Research
Date Submitted by the Author:	14-Jan-2019
Complete List of Authors:	Williamson, Sian; University of Warwick, Health Sciences<br>Johnson, Rebecca; Coventry University, Public Health<br>Sandhu, Harbinder; University of Warwick Warwick Medical School, Clinical Trials Unit<br>Jenkins, Jacquie; Public Health England, National Programme Manager – NHS Breast Screening Programme<br>Casey, Margaret; Royal Wolverhampton NHS Trust, Clinical Nurse Specialist Breast Care<br>Kearins, Olive; Public Health England, National Lead Breast Screening QA<br>Taylor-Phillips, Sian; University of Warwick, Warwick Medical School
Keywords:	PREVENTIVE MEDICINE, Health policy < HEALTH SERVICES ADMINISTRATION & MANAGEMENT, QUALITATIVE RESEARCH, Quality in health care < HEALTH SERVICES ADMINISTRATION & MANAGEMENT

SCHOLARONE™
Manuscripts

V.8 14.01.2019

Back to top

Telephone results services in English breast screening centres: a survey of current communication trends and issues

Author list: Sian Williamson (PhD Student, University of Warwick), Rebecca Johnson (Senior Lecturer in Public Health, Coventry University), Harbinder Sandhu (Associate Professor, University of Warwick), Jacquie Jenkins (National Programme Manager – NHS Breast Screening Programme, Royal Wolverhampton NHS Trust), Margaret Casey (Clinical Nurse Specialist Breast Care), Olive Kearins (National Lead Breast Screening QA, Public Health England) & Sian Taylor-Phillips (Associate Professor, University of Warwick).

Corresponding author: Rebecca Johnson,

Senior Lecturer in Public Health
Coventry University
Becky.Johnson@Coventry.ac.uk

Word count (excluding abstract): 3,881 words

For peer review only - http://bmjopen.bmj.com/site/about/guidelines.xhtml

V.8 14.01.2019

Abstract

Background: Anxiety caused by false-positive results is one of the main harms associated with screening (1-3). To minimise anxiety, results need to be effectively communicated. However, there is no data for how breast screening centres in England communicate results and a lack of evidence about what impact this communication may have on women attending screening. Without knowing current communication practices, the scale of the problem remains unknown.

Objective The objective of this study was to record how breast screening centres in England deliver results from screening including both non-cancer and cancer results

Design: An online survey was sent to staff at all 77 breast screening centres in England between 2nd June 2017 and 28th February 2018. A combination of quantitative and qualitative data were collected. Quantitative data were analysed using descriptive statistics and chi-square. Qualitative data were analysed using thematic analysis.

Results 64 out of 77 breast screening centres responded for an overall response rate of 83%. When delivering non-cancer results, 12.5% of centres never telephoned results, 37.5% occasionally telephoned results and 50% routinely telephoned results. Chi-square tests revealed no regional trends in the use of telephone results services and no time advantage for centres who telephoned results routinely.

Thematic analysis revealed two key themes: managing telephone results and valuing the patient perspective.

Conclusions Delivering non-cancer results by telephone has become common in English breast screening centres. The main finding from this study was the anxiety associated with receiving results by an unexpected communication method. This is an important and modifiable factor in the communication of screening results. More

For peer review only - http://bmjopen.bmj.com/site/about/guidelines.xhtml

V.8 14.01.2019

research is needed to fully understand what impact telephone results services and variations in the results giving process might have on women attending screening. Further research could also consider how women themselves might prefer to receive their results from screening.

Article summary

Strengths and limitations

- This study gives an up-to-date picture of current results-giving practice of NHSBSP centres nationally.
- A high response rate was achieved, indicating that this is an important issue for staff working within the screening programme.
- The qualitative analysis of NHS staff perspectives allows for expansion of the intricacies of the telephone results process and the implementation concerns that NHS staff find most important.
- Further research is needed to explore whether telephone results are a quicker method for delivering results

For peer review only - http://bmjopen.bmj.com/site/about/guidelines.xhtml

V.8 14.01.2019

Telephone results services in English breast screening centres: current communication trends and issues

Background

Breast cancer impacts 'over 1.5 million women each year' globally (4). The NHS Breast Screening Programme (NHSBSP) was launched to aid the early detection of breast cancer at the population level, because early detection is linked with better prognosis (1). In the UK, over 2 million women attend breast screening every year (5).

In recent years, the NHSBSP has been considering which communication method might be preferable for delivering non-cancer results from screening. Previous guidelines recommended in-person communication as the 'gold standard'. However, in 2017-18, the NHSBSP service specification was updated to facilitate the delivery of non-cancer results by telephone (6).

There is debate about whether delivering a non-cancer result by telephone may have a negative impact on screened women. The original concern about communicating non-cancer results by telephone arose at a Breast Care Nurses meeting, prior to the policy update in 2017-18 (7). Breast Care Nurses and clinicians noticed that the communication methods used differed between their services, with some centres delivering non-cancer results by telephone and others delivering non-cancer results in-person. At the time, service specification guidelines did not recommend that non-cancer results should be delivered by telephone. Despite the recent policy update in 2017-18, Breast Care Nurses remain concerned that telephone results may impact negatively upon screened women, particularly with regards to how anxious women might feel.

Anxiety caused by false-positive results is one of the main harms associated with breast screening and has been linked to how well screening attendees understand their result (8-10). To foster attendee understanding and minimise anxiety, results need to be effectively communicated in a way that is understandable and in-line with the patients expectations (11). Telephone and face-to-face communication allow the attendee to clarify and ask questions, which increases understanding in comparison to receiving results in writing (12). However, communicating results over the

For peer review only - http://bmjopen.bmj.com/site/about/guidelines.xhtml

telephone does not capture non-verbal communication which may have a negative impact on attendee understanding (13). A further potential advantage of delivering results via telephone is that it may be quicker than in-person communication, reducing the amount of time women spend anxiously waiting for results (14). This has not yet been investigated in breast cancer screening.

There is no current record of how breast screening centres in England deliver results from screening. This is concerning due to the lack of current evidence about the impact of telephone results on women attending screening. Without knowing current communication practice, the scale of the problem remains unknown.

In this study, we aimed to record how breast screening centres in England deliver results from screening and answer the following questions:

1) How often are telephone results delivered and by whom? Does this differ when results are non-cancer versus cancer?
2) Is there a time difference between results delivered by telephone versus results delivered in-person?
3) What views, ideas or concerns do breast screening centres have about telephoning results?

For peer review only - http://bmjopen.bmj.com/site/about/guidelines.xhtml

V.8 14.01.2019

Methods

Participants

A link to an online survey hosted by the Bristol Online Survey platform was sent to all 77 breast screening centres in England between 2nd June 2017 and 28th February 2018.

The survey link was distributed to the manager of each breast screening centre via the Quality Assurance lead for each region (South West, South East, East of England, West Midlands, East Midlands, London, North East Yorkshire & Humber & North West). The link was accompanied by a brief study description, with details regarding who was expected to complete the survey. The survey was completed by a representative member of staff from each breast screening centre.

Survey completion reminders were sent periodically by the Quality Assurance leads to nonresponding centres.

Ethical approval for the survey was obtained from the Biomedical & Scientific Research Ethics Committee at the University of Warwick (REGO-2017-1908).

Survey piloting and instrument

The survey was designed using a previous tool developed by Clinical Nurse Specialist in Breast Care (Margaret Casey) in combination with discussions with key stakeholders from the NHS Breast Screening Programme. Stakeholders involved included the programme manager for the NHSBSP, the Quality Assurance Lead for the NHSBSP, and a clinical nurse specialist in breast care. Following this, a draft version underwent three rounds of piloting: stakeholder review of content, stakeholder piloting of the online layout and cognitive interviewing with a layperson.

The main questions in the survey focused on recording how often telephoned results were used (never, occasionally or routinely) when delivering non-cancer results and when delivering cancer results. The survey also recorded who is responsible within the team for delivering telephone results at the centre (Clinical Nurse Specialist, Radiologist, Radiographer, Breast Care Surgeon, Administrative Staff, Other). After

For peer review only - http://bmjopen.bmj.com/site/about/guidelines.xhtml

V.8 14.01.2019

each question, a free text box was added to allow responders to comment on any of the answers provided.

One question recorded the amount of time taken between clinic assessment and the delivery of a result (options spanning between 1 day and more than 12 days). These data were collected to compare the length of time taken to deliver results for centres who delivered results by telephone routinely versus those who never deliver telephone results.

The survey included 9 questions. It was expected that the survey would take 10 minutes to complete. If survey responders had any questions, they were informed that they could contact the lead researcher.

Data analysis

After collection was complete, data cleaning processes were implemented. This involved checking for missing data, removing variables with no/low response rate, coding centres by region and removing duplicate responses.

Descriptive statistics for the responses were calculated. This included the response rates stratified by region.

Percentages for delivering non-cancer results and cancer results by telephone were calculated separately, alongside frequencies for who delivers each type of results.

To identify whether there were any regional trends in the delivery of telephone results, a chi-square was calculated, comparing region (South West, South East, East of England, West Midlands, East Midlands, London, North East Yorkshire & Humber & North West) with telephone frequency (routinely, occasionally, never).

To identify any potential time differences between telephone and in-person results a chi-square was calculated, comparing telephone frequency (routinely, occasionally, never) to length of time between assessment and results (1 day – 12+ days).

Statistical significance was assumed at $p<.05$. Statistical analysis was conducted using IBM SPSS Statistics 24 software.

For peer review only - http://bmjopen.bmj.com/site/about/guidelines.xhtml

Qualitative free text responses were analysed using thematic analysis following the six-step process outlined by Braun & Clarke (15). This process involves familiarisation with the data, generating initial codes, searching for themes, reviewing themes, defining and naming themes and producing the report.

Patient and Public involvement

Patients and/or public involvement was not used in this research.

For peer review only

For peer review only - http://bmjopen.bmj.com/site/about/guidelines.xhtml

V.8 14.01.2019

Results

Respondents

Of the 77 breast screening centres in England, 64 responded for an overall response rate of 83%. All regions were represented, with response rates varying between 50% and 100% for each region.

Non-cancer results by telephone

When delivering non-cancer results, 12.5% of centres never telephoned results, 37.5% occasionally telephoned results and 50% routinely telephoned results (Figure 1). When non-cancer results were delivered by telephone, the majority of these results were delivered by Clinical Nurse Specialists (n=43), followed by radiologists (n=17). No centres reported that results were delivered by administrative staff.

[INSERT FIGURE HERE]

Figure 1: Percentage of centres who deliver non-cancer results by telephone

For peer review only

Cancer results by telephone

When delivering cancer results, 75% of centres never telephoned results and 25% of centres occasionally telephoned results. No centres reported delivering cancer results routinely by telephone. When cancer results were delivered by telephone, the majority of these results were delivered by Clinical Nurse Specialists (n=14), followed by radiologists (n=2) and breast care surgeons (n=2).

Regional differences – frequency of telephone results

A chi-square test was performed and no relationship was found between region and the frequency of non-cancer telephone results, X^2 (14, N = 64) = 10.88, p =.70. This indicates no regional trends in the use of telephone results services.

Time difference – telephone results vs. in-person results

A chi-square test was performed and no relationship was found between frequency of telephone results and length of time from assessment to receipt of results, X^2 (16, N = 64) = 12.90, p =.68. This indicates no time difference for centres delivering results by telephone, suggesting that telephone results might not be quicker than in-person appointments.

For peer review only - http://bmjopen.bmj.com/site/about/guidelines.xhtml

V.8 14.01.2019

Qualitative findings – thematic analysis

In the survey, NHS staff had the opportunity to comment in free-text boxes after the two questions addressing the frequency of telephone results, when the result was non-cancer or cancer. 31 responses were recorded for delivering non-cancer results. 23 responses were recorded for delivering cancer results. 31 out of 64 centres made comments in at least one of the available free-text boxes, with a total of 48% of the sampled centres making comments. 17 out of 64 centres made comments in both free-text boxes.

34 codes were initially identified. These were condensed into 7 categories: the process of delivering results, unexpected telephone results, quality assurance and policy, setting, patient value, patient choice and managing uncertainty/anxiety. These categories were then refined further into themes.

Two key themes were identified from the thematic analysis of this data:

- Theme 1 – Managing telephone results: process and preparation
- Theme 2 – Valuing the patient perspective

For an in-depth presentation of these themes, see Appendix 1.

For peer review only

For peer review only - http://bmjopen.bmj.com/site/about/guidelines.xhtml

V.8 14.01.2019

Theme 1 – Managing telephone results: process and preparation

The first theme illustrates comments made by breast screening staff relating to the management of telephone results. This includes differences within the process of delivering telephone results (including routine telephone results services and expected versus unexpected telephone calls) and how women are prepared for the way in which their results will be communicated.

Routine telephone results

Despite the apparent frequency of telephone results services nationally, as identified in the quantitative part of the survey, there were some comments by NHSBSP staff about how, in their service, telephone results are not used.

"Standard practice is all screening patients receive an appointment for results, benign or malignant. Rarely are these communicated by telephone." (West Midlands)

This is in direct contrast to the high number of centres who responded to the survey, stating that they routinely telephone non-cancer results. It is evident that there is still a divide between best practice guidelines and what is happening in practice.

Qualitative findings revealed that, when centres deliver a cancer result by telephone, this was only at the patients request and under extreme circumstances where the results could not be delivered in-person.

Telephone results processes: expected versus unexpected

For centres who do deliver non-cancer results by telephone, it became clear that there are two different processes: expected and unexpected telephone results. Expected telephone results are received by the woman telephoning her centre or being telephoned, as prearranged at her assessment visit.

"Women call in to us for the result" (South West)

Unexpected telephone results involve giving the woman an appointment to return in-person for her result. Following a multidisciplinary team meeting, if the woman has a non-cancer result, she will then be telephoned ahead of her pre-arranged

For peer review only - http://bmjopen.bmj.com/site/about/guidelines.xhtml

V.8 14.01.2019

appointment to be told her result by telephone. She will not know she will be telephoned with the result until the moment the conversation occurs.

"Our aim is to call all the benign results and offer to cancel the booked appointment." (East Midlands)

Preparedness

Both of these different processes (expected and unexpected telephone results) have issues relating to how the woman is prepared for her result. For expected telephone results, a woman would expect to be told her result over the phone. However, some centres will not deliver cancer results by telephone in these circumstances. Therefore, when the telephone contact is made, the woman is then told that she needs to come back for her results in-person.

'If there is a positive result which was unexpected a Breast Care Nurse rings the woman to advise an appointment is required to discuss the results' (North East Yorkshire & Humber)

This may increase anxiety for the woman, who may now anticipate a potential cancer result. If there had been no expectation of a telephone result, then the woman could have been informed in-person only, thus potentially minimising her anxiety.

A similar problem occurs for unexpected telephone results, but for different reasons. Some centres 'do not inform' (West Midlands) women that they may telephone with results if they are non-cancer. This lack of awareness of how the results will be delivered contributes to the anxiety of receiving an unexpected telephone call. Women may feel unprepared and not in the right mind-set to comprehend the information given or ask questions when a telephone result was not the expected communication method.

In breast screening, it is difficult to anticipate whether the result will be non-cancer or cancer until the result has been analysed by pathology. Therefore, it is not always possible to fully prepare women for what result they might receive. Although the content of the result itself cannot be anticipated, the woman can still be prepared for the way in which results will be communicated. Many staff identified the need to

For peer review only - http://bmjopen.bmj.com/site/about/guidelines.xhtml

prepare women for the results process, and how they might feel receiving a result by telephone, in order to help manage uncertainty and minimise anxiety.

"It is always explained that the result maybe different than expected and how they would feel about that." (South West)

"They are asked if the result was a surprise and was a breast cancer, would you still wish to get that news over the phone." (South West)

This theme reveals that the communication method used may not contribute to anxiety in isolation. Instead, this may be linked to how well prepared the woman feels to receive her result, regardless of the modality.

For peer review only

V.8 14.01.2019

Theme 2 – Valuing the patient perspective

The second theme illustrates comments made by breast screening staff relating to valuing the patient perspective. This included comments relating to the setting where results are received, the convenience of results communication methods and giving women a choice.

Setting

Staff identified that women should feel comfortable and be where they would prefer to be when receiving results.

"So they are where they want to be to get the result." (South West)

Despite identifying this as an issue, staff did not address different reasons why women might prefer to receive results in a particular setting. For some women, receiving a telephone result in the comfort of their own home might be how they would prefer to be contacted. This could be due to several factors such as removing the stress of travel and parking. Some women might even have a fear of the hospital environment which might increase their anxiety.

However, other women might prefer to attend the centre in-person. For these women, the hospital environment might be reassuring, with women feeling like they are in knowledgeable and capable hands. Some women might prefer attending the centre to receive results in person, feeling a higher-level of support from a face-to-face interaction.

Convenience

Several members of staff wrote about how receiving a result in-person might be inconvenient for some women. Many women have 'issues returning', which may be due to 'considerable health problems' or being 'away for an extended period of time'.

"It would be inconvenient for the women to return in-person". (East Midlands)

It appears that these factors may act as a justification for centres currently using telephone results services, despite the lack of evidence and research into the patient impact of telephone results. Staff reported feeling as though they are valuing the

For peer review only - http://bmjopen.bmj.com/site/about/guidelines.xhtml

V.8 14.01.2019

patient perspective and providing results services in-line with women's preferences. This is reflected in various unpublished audits of individual telephone results services, which appear to show a high level of satisfaction.

Choice

NHS staff commented on how they strive to provide screening attendees with a choice about how they would like to hear their results.

"Women are given a choice." (East Midlands)

"Women are asked at assessment if they would like a telephone call or they can come back for results if they do not wish to be telephoned." (East of England)

Another aspect of 'choice' that was discussed was giving women a choice to still attend in-person when their result was delivered by telephone. This allows women who are feeling uncertain at the time of the telephone result to attend a further appointment in-person if they require 'further information/support'. Staff commented that responses to this choice vary: in some cases 'some [women] do' attend a further appointment, but in other cases 'no one ever does' take the opportunity to return in-person.

For peer review only

V.8 14.01.2019

Discussion

The method of communication for delivering results in breast screening differs between centres, which also appears to be the case in other screening programmes (16). In summary, half of the centres surveyed routinely deliver non-cancer results by telephone in contrast to a small percentage who never do. The majority of cancer results are delivered in-person, with telephone results only arranged at the patient's request. There appears to be no time advantage for centres who routinely deliver results by telephone versus those who never do, which contradicts articles citing 'speed' as one of the potential advantages of telephone results (13). However, qualitative analysis of the data revealed a variety of other potential differences, issues and patient values.

Despite the frequency of delivering non-cancer results by telephone, the qualitative data revealed this is not routine practice for all centres. For centres delivering non-cancer results by telephone, two different processes appear to be used: Expected telephone results and non-expected telephone results.

Expected telephone results might minimise anxiety for women who do not have cancer, because they have their result delivered in-line with their expectation. However, if the woman is then called in for a result, this leads to worry in anticipation of a cancer result. This might be due to some women anticipating the worst when asked to attend the clinic in-person (10, 17). Conversely, giving all women a result to attend in-person and only telephoning women with a benign result removes this issue. However, because a telephone result is unexpected in this case, this lack of awareness of how the results will be delivered is likely to contribute to anxiety, leaving women confused about their result and having unanswered questions.

Preparedness was a theme that emerged strongly from the data. In other breast screening research, women who felt well prepared for the possibility of recall experienced lower levels of distress if they were recalled (18). Research has found a clear association between uncertainty and higher levels of anxiety (2, 19-21). Uncertain information is frequently appraised by individuals as more worrying than information that is clearly positive or negative (22). This suggests that consideration should be given to what impact an unexpected method of receiving results might have on anxiety in breast screening, particularly due to the uncertainty associated

For peer review only - http://bmjopen.bmj.com/site/about/guidelines.xhtml

with a false-positive result (23). One potential way to minimise this type of anxiety would be to minimise the ambiguity associated with the results communication process (24, 25).

Patient choice was another theme that emerged from the data. However, there are feasibility issues surrounding giving all patients a choice (26, 27). Centres already delivering results by telephone may have reduced capacity to provide results in-person, with telephone clinics taking up less staff time than in-person clinics. Allowing all patients a choice could put pressure on the centre in terms of time and staffing at the organisational level. Instead, some centres do not offer patients a choice in the method of communication they receive. They telephone the woman but still offer her the opportunity to attend the clinic to discuss this further in-person. In practice, this appears to make women feel reassured enough that they then do not feel the need to attend. This approach makes women feel valued without putting a strain on staffing or resources.

Several of the comments written by NHS staff focused on patient values when considering how results should be communicated. NHS focused issues such as the potential time saving or cost savings of telephone results services were not mentioned (28). The focus remained on the women attending and valuing the patient perspective with patient values used to rationalise the use of current telephone results services. Research from the patient perspective would be beneficial, to see what issues the women themselves raise about results communication, whether these issues are concordant or discordant with the views of NHS staff and how these views may shape future communication in NHS breast screening services.

For peer review only

V.8 14.01.2019

Strengths

This study gives an up-to-date picture of current results-giving practice of NHSBSP centres nationally. A high response rate was achieved, indicating that this is an important issue for staff working within the screening programme (29). The qualitative analysis of NHS staff perspectives allows for expansion of the intricacies of the telephone results process and the implementation concerns that NHS staff find most important.

Limitations

The time difference between telephone results and in-person results was quantified in a way to allow the survey to be easily answered. This was quantified by the difference in days between clinic assessment and the delivery of a result for centres who either routinely, occasionally or never telephone results. The differences seen in process between the centres indicate that differences in speed of results delivery might not always be related to a specific method. However, a more in-depth exploration of time difference would give a better indication of whether i women who receive telephone results receive these quicker than in-person results.

For peer review only

For peer review only - http://bmjopen.bmj.com/site/about/guidelines.xhtml

V.8 14.01.2019

Conclusion

Delivering non-cancer results by telephone has become a common trend in English breast screening centres. Important issues for staff included the management of telephone results and valuing the patient perspective. The main finding from this study was the differences within telephone results services, with some centres delivering expected telephone results whilst others deliver unexpected telephone results. The anxiety that may be associated with results delivery might not be influenced by communication method alone, with the role of expected versus unexpected communication playing a key role. This is an important and modifiable factor in the communication of screening results.

Now that we have a record of current practice, more research is needed in order to fully understand what impact telephone results services have on women attending screening, and whether variations in the results giving process also have an impact. Further research could also consider how women themselves might prefer to receive their results from screening, with several patient values identified by NHS staff when considering results communication methods.

For peer review only

For peer review only - http://bmjopen.bmj.com/site/about/guidelines.xhtml

V.8 14.01.2019

Funding statement

This research is part of a PhD award and is funded by the Economic and Social Research Council (ESRC) Doctoral Training Centre at the University of Warwick. The funding has been awarded for this studentship to Sian Williamson for her PhD project for 4 years of full-time study. The award consists of payment of academic fees and a maintenance award.

A further contract between the University of Warwick, Public Health England (PHE) and the PhD Student (Sian Williamson) has secured £4,000 in research expenses.

For peer review only

Contributor ship statement

SZW is the lead researcher. SZW and RJ drafted the manuscript. SZW, RJ, HS, STP, JJ, MC and OK participated in the design of the study. SZW collected and analysed the data. OK and JJ assisted with the dissemination of the survey. All authors have reviewed and approved the manuscript.

Competing interests

None declared.

Data sharing statement

All data relevant to the study are included in the article.

V.8 14.01.2019

References

1. Keyzer-Dekker CM, De Vries J, van Esch L, Ernst MF, Nieuwenhuijzen GA, Roukema JA, et al. Anxiety after an abnormal screening mammogram is a serious problem. The Breast. 2012;21(1):83-8.
2. Liao M-N, Chen M-F, Chen S-C, Chen P-L. Uncertainty and anxiety during the diagnostic period for women with suspected breast cancer. Cancer nursing. 2008;31(4):274-83.
3. Consedine NS, Magai C, Krivoshekova YS, Ryzewicz L, Neugut AI. Fear, anxiety, worry, and breast cancer screening behavior: a critical review. Cancer Epidemiology and Prevention Biomarkers. 2004;13(4):501-10.
4. WHO. Breast cancer 2018 [cited 2018 5th March 2018]. Available from: http://www.who.int/cancer/prevention/diagnosis-screening/breast-cancer/en/.
5. UK CR. Breast screening 2017 [cited 2018 5th March]. Available from: http://www.cancerresearchuk.org/about-cancer/breast-cancer/screening/breast-screening.
6. England. PH. NHS public health functions agreement 2017- 18 Service specification no.24 Breast Screening Programme. In: England. PH, editor. 2017.
7. Liston J, Wilson R, Cooke J. Quality assurance guidelines for breast cancer screening radiology. NHSBSP publication. 2005(59).
8. Bond M, Pavey T, Welch K, Cooper C, Garside R, Dean S, et al. Systematic review of the psychological consequences of false-positive screening mammograms. 2013.
9. Brewer NT, Salz T, Lillie SE. Systematic Review: The Long-Term Effects of False-Positive MammogramsThe Long-Term Effects of False-Positive Mammograms. Annals of internal medicine. 2007;146(7):502-10.
10. Bolejko A, Zackrisson S, Hagell P, Wann-Hansson C. A roller coaster of emotions and sense–coping with the perceived psychosocial consequences of a false-positive screening mammography. Journal of clinical nursing. 2014;23(13-14):2053-62.
11. Ong LM, De Haes JC, Hoos AM, Lammes FB. Doctor-patient communication: a review of the literature. Social science & medicine. 1995;40(7):903-18.
12. Gurol-Urganci I, de Jongh T, Vodopivec-Jamsek V, Car J, Atun R. Mobile phone messaging for communicating results of medical investigations. Cochrane Database of Systematic Reviews. 2008(4).
13. Car J, Sheikh A. Telephone consultations. Bmj. 2003;326(7396):966-9.
14. Heleno B, Siersma V, Brodersen J. Waiting time and the psychosocial consequences of false-positive mammography: cohort study. Journal of negative results in biomedicine. 2015;14(1):8.
15. Braun V, Clarke VJQrip. Using thematic analysis in psychology. 2006;3(2):77-101.
16. Philips Z, Johnson S, Avis M, Whynes D. Communicating mild and borderline abnormal cervical smear results: how and what are women told? Cytopathology. 2002;13(6):355-63.
17. Solbjør M, Forsmo S, Skolbekken J-A, Sætnan AR. Experiences of recall after mammography screening—a qualitative study. Health care for women international. 2011;32(11):1009-27.
18. Austoker J, Ong GJJoMS. Written information needs of women who are recalled for further investigation of breast screening: results of a multicentre study. 1994;1(4):238-44.

For peer review only - http://bmjopen.bmj.com/site/about/guidelines.xhtml

V.8 14.01.2019

19. Skotko BG, Kishnani PS, Capone GT, A DSDSGJAjomgP. Prenatal diagnosis of Down syndrome: How best to deliver the news. 2009;149(11):2361-7.
20. Sweeny K, Cavanaugh AG. Waiting is the hardest part: A model of uncertainty navigation in the context of health news. Health Psychology Review. 2012;6(2):147-64.
21. Marteau TMJBBMJ. Screening in practice: Reducing the psychological costs. 1990;301(6742):26.
22. Sweeny K, Reynolds CA, Falkenstein A, Andrews SE, Dooley MD. Two definitions of waiting well. Emotion. 2016;16(1):129.
23. Fallowfield L, Jenkins V. Effective communication skills are the key to good cancer care. European Journal of Cancer. 1999;35(11):1592-7.
24. Brett J, Austoker J. Women who are recalled for further investigation for breast screening: psychological consequences 3 years after recall and factors affecting re-attendance. Journal of Public Health. 2001;23(4):292-300.
25. Mayou RJTBJoGP. Screening in primary care: pointers for further research. 1996;46(411):567.
26. Appleby J, Harrison A, Devlin NJ. What is the real cost of more patient choice?: King's Fund London; 2003.
27. O'Connell R, Hartridge-Lambert S, Din N, St John E, Hitchins C, Johnson T. Patients' understanding of medical terminology used in the breast clinic. The Breast. 2013;22(5):836-8.
28. Brazeal HA, Holley SO, Appleton CM, Lee MVJTbj. Patient preferences for breast biopsy result notification. 2018.
29. McColl E, Jacoby A, Thomas L, Soutter J, Bamford C, Steen N, et al. Design and use of questionnaires: a review of best practice applicable to surveys of health service staff and patients: Core Research; 2001.

For peer review only

For peer review only - http://bmjopen.bmj.com/site/about/guidelines.xhtml

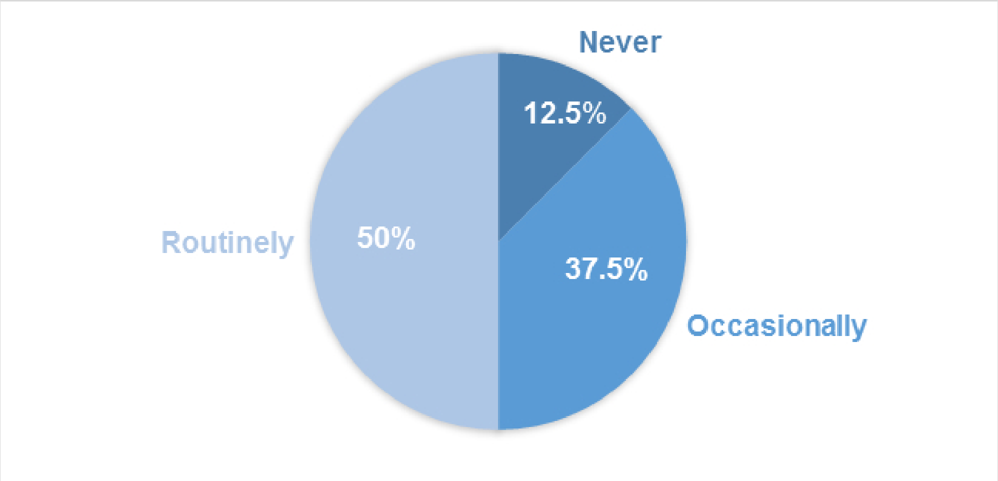

Figure 1: Percentage of centres who deliver non-cancer results by telephone

Theme	Sub-theme	Example quotes
**THEME 1 Managing telephone results: process and preparation**	**Routine telephone results**	“This is not routine practice but happens if a patient requests it and the probability of a benign result is very high.” (North West)<br>“Standard practice is all screening patients receive an appointment for results, benign or malignant. Rarely are these communicated by telephone.” (West Midlands)<br>“This is not done routinely and very rarely occurs” (North West)
	**Telephone results processes: expected versus unexpected**	**EXPECTED**<br>“Call in to us for a result as prearranged” (South West)<br>“Women call in to us for the result”. (South West)<br><br>‘If there is a positive result which was unexpected a Breast Care Nurse rings the woman to advise an appointment is required’ (North East Yorkshire & Humber)<br>‘Women are only telephoned with a cancer result if they have specifically asked for it that way and signed to give their consent’ (South West)<br><br>**UNEXPECTED**<br>‘Scheduled face to face appt. for results but if its benign we ring them.’ (South West)<br>‘Our aim is to call all the benign results and offer to cancel the booked appointment’ (East Midlands)<br>‘All women are given a results appointment during assessment clinic. Following MDT, those with benign biopsy results are contacted by telephone’ (West Midlands)<br><br>‘Women are seen in the assessment clinic and are asked by the BCN for permission to ring if need be to alter appts etc but we do not inform them that we will with the results in case it is more appropriate for them to come to clinic to receive the results.’ (Dudley, Wolverhampton & South West)
	**Preparedness**	‘If there is a positive result which was unexpected a Breast Care Nurse rings the woman to advise an appointment is required’ (North East Yorkshire & Humber)<br>‘Women are only telephoned with a cancer result if they have specifically asked for it that way and signed to give their consent’ (South West)<br><br>‘Women are seen in the assessment clinic and are asked by the BCN for permission to ring if need be to alter appts etc but we do not inform them that we will with the results in case it is more appropriate for them to come to clinic to receive the results.’ (Dudley, Wolverhampton & South West)<br><br>“It is always explained that the result maybe different than expected and how they would feel about that.” (South West)<br><br>“They are asked if the result was a surprise and was a breast cancer, would you still wish to get that news over the phone.” (South West)

Theme	Sub-theme	Example quotes
THEME 2: Valuing the patient perspective	Setting	'So they are where they want to be to get the result' (South West)<br>'Most women do not wish to come to clinic' (East of England)
	Convenience	'It would be inconvenient for the women to return in person' (East Midlands)<br>'Only rung if they have issues returning' (East of England)<br>'Will phone them if they are unable to attend their appointment' (East of England)<br>'Very difficult for woman to attend' (North East Yorkshire & Humber)<br>'If a woman is to be away for an extended period of time' (West Midlands)<br>'If the patient has considerable health problems which attending hospital makes very difficult' (East Midlands)
	Choice	'Women are given a choice' (East Midlands)<br>'Women are asked at assessment if they would like a telephone call or they can come back for results if they do not wished to be telephoned' (East of England)<br>They are asked if the result was a surprise and was a breast cancer would you still wish to get that news over the phone (South West)<br>'We always stress they can come in anyway. No one ever does!' (East Midlands)<br>'Patients are still able to attend, and some do.' (East Midlands)<br>'Are still offered to come to see a clinician if they still wish' (East of England)<br>'Unless the woman specifically wants to attend for further information/support' (West Midlands)

Reporting checklist for cross sectional study.

Based on the STROBE cross sectional guidelines.

Instructions to authors

Complete this checklist by entering the page numbers from your manuscript where readers will find each of the items listed below.

Your article may not currently address all the items on the checklist. Please modify your text to include the missing information. If you are certain that an item does not apply, please write "n/a" and provide a short explanation.

Upload your completed checklist as an extra file when you submit to a journal.

In your methods section, say that you used the STROBE cross sectional reporting guidelines, and cite them as:

von Elm E, Altman DG, Egger M, Pocock SJ, Gotzsche PC, Vandenbroucke JP. The Strengthening the Reporting of Observational Studies in Epidemiology (STROBE) Statement: guidelines for reporting observational studies.

		Reporting Item	Page Number
Title	#1a	Indicate the study's design with a commonly used term in the title or the abstract	1
Abstract	#1b	Provide in the abstract an informative and balanced summary of what was done and what was found	2

For peer review only

Background / rationale	#2	Explain the scientific background and rationale for the investigation being reported	4
Objectives	#3	State specific objectives, including any prespecified hypotheses	5
Study design	#4	Present key elements of study design early in the paper	6
Setting	#5	Describe the setting, locations, and relevant dates, including periods of recruitment, exposure, follow-up, and data collection	6
Eligibility criteria	#6a	Give the eligibility criteria, and the sources and methods of selection of participants.	6
	#7	Clearly define all outcomes, exposures, predictors, potential confounders, and effect modifiers. Give diagnostic criteria, if applicable	n/a
Data sources / measurement	#8	For each variable of interest give sources of data and details of methods of assessment (measurement). Describe comparability of assessment methods if there is more than one group. Give information separately for for exposed and unexposed groups if applicable.	n/a
Bias	#9	Describe any efforts to address potential sources of bias	7
Study size	#10	Explain how the study size was arrived at	6

For peer review only

Quantitative variables	#11	Explain how quantitative variables were handled in the analyses. If applicable, describe which groupings were chosen, and why	7
Statistical methods	#12a	Describe all statistical methods, including those used to control for confounding	7
	#12b	Describe any methods used to examine subgroups and interactions	7
	#12c	Explain how missing data were addressed	7
	#12d	If applicable, describe analytical methods taking account of sampling strategy	n/a
	#12e	Describe any sensitivity analyses	n/a
Participants	#13a	Report numbers of individuals at each stage of study—eg numbers potentially eligible, examined for eligibility, confirmed eligible, included in the study, completing follow-up, and analysed. Give information separately for for exposed and unexposed groups if applicable.	9
	#13b	Give reasons for non-participation at each stage	n/a
	#13c	Consider use of a flow diagram	n/a
Descriptive data	#14a	Give characteristics of study participants (eg demographic, clinical, social) and information on exposures and potential confounders. Give information separately for exposed and unexposed groups if applicable.	9

For peer review only

	#14b	Indicate number of participants with missing data for each variable of interest	n/a
Outcome data	#15	Report numbers of outcome events or summary measures. Give information separately for exposed and unexposed groups if applicable.	9
Main results	#16a	Give unadjusted estimates and, if applicable, confounder-adjusted estimates and their precision (eg, 95% confidence interval). Make clear which confounders were adjusted for and why they were included	n/a
	#16b	Report category boundaries when continuous variables were categorized	n/a
	#16c	If relevant, consider translating estimates of relative risk into absolute risk for a meaningful time period	n/a
Other analyses	#17	Report other analyses done—e.g., analyses of subgroups and interactions, and sensitivity analyses	n/a
Key results	#18	Summarise key results with reference to study objectives	17
Limitations	#19	Discuss limitations of the study, taking into account sources of potential bias or imprecision. Discuss both direction and magnitude of any potential bias.	19
Interpretation	#20	Give a cautious overall interpretation considering objectives, limitations, multiplicity of analyses, results from similar studies, and other relevant evidence.	20

For peer review only

Generalisability	#21	Discuss the generalisability (external validity) of the study results	20
Funding	#22	Give the source of funding and the role of the funders for the present study and, if applicable, for the original study on which the present article is based	21

The STROBE checklist is distributed under the terms of the Creative Commons Attribution License CC-BY. This checklist was completed on 19. December 2018 using http://www.goodreports.org/, a tool made by the EQUATOR Network in collaboration with Penelope.ai

For peer review only

BMJ Open

Communicating biopsy results from screening assessment: Current practice in English breast screening centres and staff perspectives of telephoning results

Journal:	*BMJ Open*
Manuscript ID	bmjopen-2018-028683.R1
Article Type:	Original research
Date Submitted by the Author:	17-May-2019
Complete List of Authors:	Williamson, Sian; University of Warwick, Health Sciences<br>Johnson, Rebecca; Coventry University<br>Sandhu, Harbinder; University of Warwick Warwick Medical School, Clinical Trials Unit<br>Ellard, David; Warwick Medical School, Clinical trials Unit<br>Jenkins, Jacquie; Public Health England, National Programme Manager – NHS Breast Screening Programme<br>Casey, Margaret; Royal Wolverhampton NHS Trust, Clinical Nurse Specialist Breast Care<br>Kearins, Olive; Public Health England, National Lead Breast Screening QA<br>Taylor-Phillips, Sian; University of Warwick, Warwick Medical School
**Primary Subject Heading**:	Communication
Secondary Subject Heading:	Health policy, Health services research, Oncology, Public health
Keywords:	PREVENTIVE MEDICINE, Health policy < HEALTH SERVICES ADMINISTRATION & MANAGEMENT, QUALITATIVE RESEARCH, Quality in health care < HEALTH SERVICES ADMINISTRATION & MANAGEMENT

SCHOLARONE™
Manuscripts

V.9 23.04.2019

Back to top

Communicating biopsy results from screening assessment: Current practice in English breast screening centres and staff perspectives of telephoning results

Author list: Sian Williamson (PhD Student, University of Warwick), Rebecca Johnson (Senior Lecturer in Public Health, Coventry University), Harbinder Sandhu (Associate Professor, University of Warwick), David Ellard (Associate Professor, University of Warwick), Jacquie Jenkins (National Programme Manager – NHS Breast Screening Programme, Royal Wolverhampton NHS Trust), Margaret Casey (Clinical Nurse Specialist Breast Care), Olive Kearins (National Lead Breast Screening QA, Public Health England) & Sian Taylor-Phillips (Associate Professor, University of Warwick).

Corresponding author: Rebecca Johnson,
Coventry University
ad1658@coventry.ac.uk

Word count (excluding abstract and tables): 4,118 words

For peer review only - http://bmjopen.bmj.com/site/about/guidelines.xhtml

V.9 23.04.2019

Abstract (287 words)

Objective

To record how breast screening centres in England deliver all biopsy results (cancer/non-cancer) from the screening assessment visit.

Design

Online survey of 63 of 79 breast screening centres in England from all regions (East Midlands, East of England, London, North East Yorkshire & Humber, North West, South East, South West, West Midlands). The survey contained quantitative measures of frequency for telephoning biopsy results (routinely, occasionally or never) and qualitative free-text responses. Surveys were completed by a staff member from each centre.

Results

There were no regional trends in the use of telephone results services, (X^2 (14, N = 63) = 11.55, p =.64), Centres who telephoned results routinely did not deliver results sooner, (X^2 (16, N = 63) = 12.76, p =.69).

When delivering cancer results, 76.2% of centres never telephone results and 23.8% of centres occasionally telephone results. No centres reported delivering cancer results routinely by telephone. Qualitative comments suggest that cancer results are only telephoned at patient request and under exceptional circumstances.

When delivering non-cancer results, 12.7% of centres never telephoned results, 38.1% occasionally telephoned results and 49.2% routinely telephoned results. Qualitative comments revealed different processes for delivering telephone results, including patient choice and scheduling an in-person results appointment for all women attending screening assessment, then ringing non-cancer results unexpectedly ahead of this pre-booked appointment.

Conclusions

In the NHS Breast Screening Programme, screening assessment results that are cancer are routinely delivered in-person. However, non-cancer screening assessment results are often routinely delivered by telephone, despite policy recommendations.

More research is needed to understand the impact of telephoning results on women attending screening, particularly women who receive a non-cancer result. Future research should also consider how women themselves might prefer to receive their screening assessment results.

For peer review only - http://bmjopen.bmj.com/site/about/guidelines.xhtml

V.9 23.04.2019

Article summary

Strengths and limitations

- This study gives an up-to-date picture of current results-giving practice of NHSBSP centres nationally.
- A high response rate was achieved, so results are generalizable to the English NHS Breast Screening Programme
- The qualitative comments made by NHS staff gave deeper insight into how telephone results are delivered in practice.
- Survey responses were subjective and not checked against centre policy documents

For peer review only

For peer review only - http://bmjopen.bmj.com/site/about/guidelines.xhtml

V.9 23.04.2019

Communicating biopsy results from screening assessment: Current practice in English breast screening centres and staff perspectives of telephoning results

Background

Breast cancer is one of the most common cancers internationally (1). The National Health Service Breast Screening Programme (NHSBSP) was launched to aid the early detection of breast cancer at the population level, because early detection is linked with better prognosis (2). In the UK, over 2 million women attend breast screening every year, where they have an initial mammogram (3). If an abnormality is found during this screening mammogram, women will be recalled to attend screening assessment for further tests which aim to confirm whether the abnormality found is a cancer. These tests can include a core needle biopsy, which involves the removal of sections of tissue from the suspicious breast region which are sent for cytological examination. A biopsy is the definitive test at screening assessment to confirm if the mammogram abnormalities found are cancer. In 2014-15, 1,795,307 women in England were screened by the NHSBSP. 68,973 of these women were recalled to attend screening assessment and 31,926 women had a biopsy (4).

In recent years, the NHSBSP has been considering which communication method might be preferable for delivering non-cancer biopsy results from screening assessment. The NHSBSP service specification recommends that all screening assessment results should be delivered in-person, which includes cancer and non-cancer results (5). Furthermore, the guidelines state that telephone results should only be used at the patients request and should not be standard practice (6). Despite these recommendations, there is a suggestion that some centres routinely deliver non-cancer screening assessment results by telephone (5, 7). There is ongoing concern about the impact of delivering a non-cancer assessment result by telephone on screened women. One of the main concerns is how anxious women feel when receiving a result from screening, even when the result is non-cancer (8). Another concern is the potential for miscommunication by telephone.

In other areas of healthcare, a variety of communication methods are used to deliver results, including face-to-face consultations, telephone, letters and email. Each

For peer review only - http://bmjopen.bmj.com/site/about/guidelines.xhtml

method of communication used in results delivery presents different advantages, disadvantages and challenges in implementation. Results delivered in-person are often seen as the 'gold standard' (9). However, as technology advances, fewer healthcare results are now delivered in-person. Liederman et al. (10) stated that 'face-to-face contact is not necessary for effective communication' (pg. 52). Most commonly, results delivery is moving towards telephone and telemedicine (11). Despite this, Cochrane review evidence suggests that we still do not know enough about the impact of telephone results services on healthcare outcomes (12). This is concerning due to the rapid changes in communication that may not have been adequately evaluated.

There is no current record of how breast screening centres in England deliver biopsy results from screening assessment. Despite policy recommendations, some centres appear to routinely deliver non-cancer results by telephone. Furthermore, it is assumed that cancer results are all delivered in-person as guidelines recommend. There is a currently lack of evidence about how often telephone result are used to deliver screening assessment results (13).

In this study, we aimed to record how breast screening centres in England deliver biopsy results from screening assessment and answer the following questions:

1) How often are telephone results delivered and by whom? Does this differ when results are non-cancer versus cancer?
2) Is there a time difference between results delivered by telephone versus results delivered in-person?

For peer review only

For peer review only - http://bmjopen.bmj.com/site/about/guidelines.xhtml

V.9 23.04.2019

Methods

Participants

A link to an online survey hosted by the Bristol Online Survey platform was sent to all breast screening centres in England on 2nd June 2017. At this time, 79 breast screening units existed in England, as confirmed by a list from the Quality Assurance Lead for Breast Screening. Data collection ended on 28th February 2018.

The survey link was distributed to the manager of each breast screening centre via the Quality Assurance lead for each region (East Midlands, East of England, London, North East Yorkshire & Humber, North West, South East, South West, West Midlands). The link was accompanied by a brief study description, with details regarding who was expected to complete the survey. The survey was completed by a representative member of staff from each breast screening centre.

Survey completion reminders were sent periodically by the Quality Assurance leads to nonresponding centres.

Ethical approval for the survey was obtained from the Biomedical & Scientific Research Ethics Committee at the University of Warwick (REGO-2017-1908).

Survey piloting and instrument

The survey was designed using a previous tool developed by Clinical Nurse Specialist in Breast Care (Margaret Casey) in combination with discussions with key stakeholders from the NHS Breast Screening Programme. Stakeholders included the programme manager for the NHSBSP, the Quality Assurance Lead for the NHSBSP, and a clinical nurse specialist in breast care. Following this, a draft version underwent three rounds of piloting: stakeholder review of content, stakeholder piloting of the online layout and cognitive interviewing with a layperson.

The main questions in the survey focused on recording how often biopsy results were telephoned. The first question asked the frequency of delivering benign (non-cancer) biopsy results by telephone (never/occasionally/routinely). The second question asked the frequency of delivering cancer biopsy results by telephone

For peer review only - http://bmjopen.bmj.com/site/about/guidelines.xhtml

V.9 23.04.2019

(never/occasionally/routinely). After these two questions, a free text box was added to allow responders to comment on the answers provided. The survey also recorded who is responsible within the team for delivering telephone results at the centre (Clinical Nurse Specialist, Radiologist, Radiographer, Breast Care Surgeon, Administrative Staff, Other).

One question recorded the amount of time taken between clinic assessment and the delivery of a result (options spanning between 1 day and more than 12 days). These data were collected to compare the length of time taken to deliver results for centres who delivered results by telephone routinely versus those who never deliver telephone results.

The survey included 9 questions. It was expected that the survey would take 10 minutes to complete. If survey responders had any questions, they were informed that they could contact the lead researcher (see Appendix 1 for full survey).

Data analysis

Quantitative

Data cleaning processes were implemented. This involved checking for missing data, coding centres by region and removing duplicate responses. Descriptive statistics for the responses were calculated. This included the response rates stratified by region.

Percentages for delivering non-cancer results and cancer results by telephone were calculated separately, alongside frequencies for who delivers each type of results.

To identify whether there were any regional trends in the delivery of telephone results, a chi-square was calculated, comparing region (East Midlands, East of England, London, North East Yorkshire & Humber, North West, South East, South West, West Midlands) with telephone frequency (routinely, occasionally, never).

To identify any potential time differences between telephone and in-person results a chi-square was calculated, comparing telephone frequency (routinely, occasionally, never) to length of time between assessment and results (1 day – 12+ days).

For peer review only - http://bmjopen.bmj.com/site/about/guidelines.xhtml

Statistical significance was assumed at p<.05. Statistical analysis was conducted using IBM SPSS Statistics 24 software.

Qualitative

Qualitative free text comments were analysed using qualitative content analysis (14, 15). This approach allowed for commonalities amongst staff viewpoints to be identified and to be described narratively, in order to contextualise and expand upon the quantitative findings. The analysis was conducted by the lead author (SW) and checked by a second author (DE) to ensure that the meaning of original staff comments was retained. Any disputes in interpretation were resolved by a third author (HS).

For peer review only

For peer review only - http://bmjopen.bmj.com/site/about/guidelines.xhtml

V.9 23.04.2019

Results

Respondents

Of the 79 breast screening centres in England, 63 responded for an overall response rate of 79%. All regions were represented in the quantitative survey (see Table 1).

Table 1: Number of centres by region who responded to the survey, 1st qualitative question and 2nd qualitative question

Region	No. of centres in region	No. of survey responses	No. of responses to 1st qualitative question (non-cancer)	No. of responses to 2nd qualitative question (cancer)
East Midlands	9	7	5	3
East of England	11	10	5	2
London	6	4	0	0
North East Yorkshire & Humber	12	10	3	1
North West	11	8	2	2
South East	8	4	2	3
South West	13	11	5	6
West Midlands	9	9	6	3
Total	79	63	28	20

Data relating to the average (mean) age of women screened at each centre were removed from the data set due to 61.4% of responses being ‘I don’t know’.

For peer review only - http://bmjopen.bmj.com/site/about/guidelines.xhtml

Quantitative findings

Non-cancer results by telephone

When delivering non-cancer results, 12.7% of centres never telephoned results, 38.1% occasionally telephoned results and 49.2% routinely telephoned results. When non-cancer results were delivered by telephone, the majority of these results were delivered by Clinical Nurse Specialists (n=42), followed by radiologists (n=17). No centres reported that results were delivered by administrative staff.

Cancer results by telephone

When delivering cancer results, 76.2% of centres never telephoned results and 23.8% of centres occasionally telephoned results. No centres reported delivering cancer results routinely by telephone. When cancer results were delivered by telephone, the majority of these results were delivered by Clinical Nurse Specialists (n=13),followed by radiologists (n=2) and breast care surgeons (n=2).

Regional differences – frequency of telephone results

No relationship was found between region and the frequency of non-cancer telephone results, (X^2 (14, N = 63) = 11.55, p =.64).. This indicates no regional trends in the use of telephone results services.

Time difference – telephone results vs. in-person results

No relationship was found between frequency of telephone results and length of time from assessment to receipt of results, (X^2 (16, N = 63) = 12.76, p =.69). This indicates that centres delivering results by telephone do not deliver them sooner after the assessment visit.

For peer review only - http://bmjopen.bmj.com/site/about/guidelines.xhtml

V.9 23.04.2019

Qualitative findings – thematic analysis

In the survey, NHS staff had the opportunity to comment in free-text boxes after two questions. The first question asked the frequency of delivering benign (non-cancer) biopsy results by telephone (never/occasionally/routinely). The second question asked the frequency of delivering cancer biopsy results by telephone (never/occasionally/routinely). The results are presented in two sections, with one focusing on first question (non-cancer) and one focusing on the second question (cancer). All regions (excluding London) provided qualitative free-text responses (see Table 1).

When delivering benign (non-cancer) biopsy results are women never telephoned with results, occasionally telephoned with results or routinely telephoned with results?

This section presents the qualitative findings following the first survey question, which asked the frequency of delivering non-cancer biopsy results by telephone (never/occasionally/routinely). 29 centres recorded free-text responses following the first survey question.

7 centres commented that they arranged face to face appointments for everyone who attends screening assessment, then attempt to telephone any women who have a non-cancer result following the multidisciplinary team meeting. Example comments include:

"Women have a scheduled face to face appointment for results but if it's benign we ring them." (Centre ID 2)

"Our aim is to call all he benign results and offer to cancel the booked appointment." (Centre ID 50)

"All women are given a results appointment during assessment clinic. Following MDT, all those with benign biopsy results are contacted by telephone. If contact is made, the result is discussed and the results appointment cancelled." (Centre ID 38)

For peer review only - http://bmjopen.bmj.com/site/about/guidelines.xhtml

V.9 23.04.2019

1 centre (Centre ID 25) commented that they “normally see women face to face”, with this being their routine practice. This suggests that breast screening centres differ in how they deliver non-cancer screening assessment results.

Option to still attend

5 centres commented that, when women are contacted by telephone with a non-cancer result, they are still offered the option to attend in-person if they have further questions. Example comments include:

“All patients are given an appointment to attend for results we do telephone with results but patients are still able to attend, and some do.” (Centre ID 45)

“After the MDT. Patients are telephoned with benign results by a qualified Breast Care Nurse. They are then offered an OPA with a consultant surgeon if they have concerns.” (Centre ID 31)

Rare and exceptional circumstances

5 centres commented that non-cancer results are only delivered by telephone in exceptional circumstances. Example comments include:

“Only in exceptional circumstances” (Centre ID 59)

“This is not done routinely and very rarely occurs.” (Centre ID 78)

Reasons given for giving non-cancer results by telephone included if the woman finds it difficult to attend in-person and so the woman can be where she wants to be to receive results.

For peer review only - http://bmjopen.bmj.com/site/about/guidelines.xhtml

V.9 23.04.2019

Giving women a choice or at patient request

5 centres commented that women are asked how they would like their result to be delivered if it is not cancer. Example comments include:

"We will always offer them an appointment to come in, but the BCNs will ask if they want a telephone call at the time of assessment" (Centre ID 63)

"Women are asked at assessment if they would like a telephone call or they can come back for results if they do not wished to be telephoned" (Centre ID 29)

"Women are given a choice about how they receive their results when the imaging suggests a benign process" (Centre ID 42)

One centre commented that non-cancer results are only delivered by telephone at the request of the patient:

"This is not routine practice but happens if a patient requests it and the probability of a benign result is very high." (Centre ID 73)

Summary of non-cancer free-text comments

Comments from centres were conflicting. Some centres schedule face-to-face appointments for all women attending screening assessment, but then attempt to contact women with non-cancer results by telephone instead. However, one centre commented that delivering non-cancer results by telephone was not routine practice. Other centres commented that telephoning non-cancer results only happens under exceptional circumstances, such as the woman being unable to attend in-person.

Centres commented that women who are telephoned with results are still offered the option to attend if they have further questions. Some centres ask women how they would prefer to be contacted with their result if it is not cancer.

For peer review only

For peer review only - http://bmjopen.bmj.com/site/about/guidelines.xhtml

V.9 23.04.2019

When delivering cancer biopsy results are women never telephoned with results, occasionally telephoned with results or routinely telephoned with results?

This section presents the qualitative findings following the second survey question, which asked the frequency of delivering cancer biopsy results by telephone (never/occasionally/routinely). 21 centres recorded free-text responses following the second survey question.

4 centres made comments suggesting that cancer results are routinely delivered in-person. Example comments include:

"Cancer diagnoses are always communicated face to face." (Centre ID 39)

"[telephone results] This would never be planned." (Centre ID 9)

"All positive results or complicated cases are invited back to be given results by the Breast Surgery Team." (Centre ID 26)

Rare and exceptional circumstances

11 centres commented that cancer results are only delivered by telephone in exceptional circumstances. Example comments include:

"Rarely telephoned with a cancer diagnosis always at the patients request in extenuating circumstances." (Centre ID 45)

"This is a rare occurrence and is only agreed to with the patients prior consent on the understanding they may be receiving a cancer diagnosis." (Centre ID 11).

"Very rare - this would only happen with prior agreement if a woman is to be away for an extended period of time." (Centre ID 38)

Reasons for delivering cancer results by telephone under these exceptional circumstances were if the woman finds it difficult to attend in-person and if the woman was going to be away for an extended period of time.

For peer review only - http://bmjopen.bmj.com/site/about/guidelines.xhtml

V.9 23.04.2019

Giving women a choice or at patient request

2 centres commented that women are asked how they would like their result to be delivered if it is cancer. Example comments include:

"They are asked if the result was a surprise and was a breast cancer would you still wish to get that news over the phone." (Centre ID 1)

"Women are asked at assessment if they would like a telephone call or they can come back for results if they do not wished to be telephoned. (Centre ID 29)"

5 centres commented that cancer results are only delivered by telephone at the request of the patient. Example comments include:

"*Patient request only" (Centre ID 33)*

"Only very rarely and at patient's specific request" (Centre ID 8)

Unexpected results and a negative reaction

One centre (Centre ID 60) addressed the issue of how to deal with an unexpected result. At the screening assessment visit, this centre informs women with a high suspicion of a non-cancer result that they will have their result delivered by telephone. However, if *"If there is a positive result which was unexpected a Breast Care Nurse rings the woman to advise an appointment is required to discuss the results".*

One centre (Centre ID 15) commented about a woman who *"reacted extremely badly on telephone"* when receiving a cancer result.

For peer review only

V.9 23.04.2019

Summary of cancer free-text comments

For cancer results, face to face results are routine. Telephoning cancer results is only being offered under rare or exceptional circumstances such as when women have difficulty in attending. These telephone results are only given at the request of the patient.

Some comments were made about the potential difficulties in delivering results by telephone. One comment highlighted the negative reaction of a woman who received her cancer result by telephone. Another comment addressed the issue of how to deal with a result which was presumed to be non-cancer but turned out to be cancer.

For peer review only

For peer review only - http://bmjopen.bmj.com/site/about/guidelines.xhtml

V.9 23.04.2019

Discussion

The aim of this research was to record how breast screening centres in England deliver biopsy results from screening assessment, by assessing how often telephone results are delivered and by whom. This research also aimed to see if telephone results delivery differs when assessment results are non-cancer versus cancer. Furthermore, this research aimed to assess if there is a time difference between results delivered by telephone versus results delivered in-person.

Firstly, our research suggests that centres delivering results by telephone do not deliver them sooner after the assessment visit. This contradicts articles citing 'speed' as one of the potential advantages of telephone results.

Overall, delivering cancer results by telephone is not common practice for breast screening centres in England. Telephoning cancer results is only used in exceptional circumstances and only at the request of the patient. For example, telephone results might be used if the woman is physically unable to attend in-person (e.g. health issues or away from the country for an extended period). When cancer results are telephoned, most of the results are delivered by Clinical Nurse Specialists.

The reason why cancer results are rarely telephoned is probably due to the emotional impact of receiving a cancer diagnosis (16). The extensive literature on breaking bad news in healthcare places importance on the location where results are received to ensure no disturbances (17). This may help to explain the one comment in the current study where a centre reported the negative reaction of a woman who received a cancer result by telephone.

Overall, delivering non-cancer results by telephone is routine practice for roughly half of the breast screening centres in England, with most of the results being delivered by Clinical Nurse Specialists. This contrasts with recommendations in breast screening policy guidelines, which state that telephone results should only be used at the patients request and should not be routinely offered. Staff commented that telephone results are used when patients find it difficult to attend in-person. A potential reason that telephone results services are being used against policy recommendations is that telephone results have the potential to be time and cost-efficient. Another potential reason is that women who attend screening may prefer to be contacted by telephone when the result is not cancer.

For peer review only - http://bmjopen.bmj.com/site/about/guidelines.xhtml

V.9 23.04.2019

Another process mentioned in staff comments about delivering non-cancer results was giving women the choice between telephone and in-person. However, there may be feasibility issues surrounding giving all patients this choice (18). For centres who already routinely telephone results, there may be a reduced capacity to provide results in-person. From the comments made by staff in the current research, a compromise is to telephone all non-cancer results routinely but to also offer the option for the woman to still attend the clinic in-person.

Comments from staff revealed a common process for centres who deliver non-cancer results by telephone. This process involves giving all women who attend screening assessment an in-person appointment to return, but then telephoning women with non-cancer results ahead of this scheduled appointment. On one hand, this process has the potential to reduce the expected wait time for women to receive results, thus minimising the amount of time spent anxiously waiting (19). On the other hand, the impact of receiving an 'unexpected' communication method has not been explored in this setting. When a telephone result is not expected, it is possible that women may feel unprepared or not in the right mind-set to comprehend the information given (20). This may contribute to the anxiety associated with screening and might be an avoidable harm. However, from the current research we do not know if this is the case.

Another comment addressed the issue of how to deal with a result which was presumed to be non-cancer but turned out to be cancer. At the stage of screening assessment, it is unknown if a woman will receive a cancer or non-cancer result. However, during the screening assessment visit, clinical signs on mammogram, ultrasound or through physical palpation may indicate a higher chance of a non-cancer result. As suggested by the current study, these women may be told that they will be telephoned with their result due to the lower suspicion of cancer. However, due to the associated uncertainty, the result may still be a cancer. According to the current study, there has been a case of a woman being told she will be telephoned with her result but the telephone call is ultimately used to schedule a result in-person. This may have implications for anxiety, with the woman potentially anticipating a cancer result. Again, this might be an avoidable harm. However, from the current research we do not know if this is the case.

For peer review only - http://bmjopen.bmj.com/site/about/guidelines.xhtml

V.9 23.04.2019

Strengths

This study formally reports the national communication practice of NHSBSP centres for delivering non-cancer and cancer screening assessment results. The high response rate indicated that this is an important issue for staff working within the screening programme.

The qualitative free-text comments allowed for expansion on quantitative survey data, which gave greater insight into how telephone results were implemented in practice.

Limitations

The time difference between telephone results and in-person results was quantified in a way to allow the survey to be easily answered. This was quantified by the difference in days between clinic assessment and the delivery of a result for centres who either routinely, occasionally or never telephone results. However, other factors could be involved in the speed of results delivery.

The survey responses completed by centre staff were subjective and could not be validated by records. Furthermore, the staff member completing each survey was not recorded as part of the study. Therefore, quantitative data may not accurately reflect current communication practice due to the potential for human error or level of experience of the staff member completing the survey.

It is possible that centres who did not respond to the survey may be systematically different from centres who did. Therefore, the results may not be representative of all breast screening centres in England. However, the high response rate limits this issue.

For peer review only - http://bmjopen.bmj.com/site/about/guidelines.xhtml

V.9 23.04.2019

Conclusion

In the NHS Breast Screening Programme, screening assessment results that are cancer are routinely delivered in-person, as recommended by policy guidelines. However, non-cancer screening assessment results are often routinely delivered by telephone despite the recommendations made in policy guidelines. Despite this, telephone results do not appear to be quicker than in-person results in practice.

The most common process is to give all women attending screening assessment an appointment to come back to receive results in-person, to then telephone all women with non-cancer results ahead of this appointment. Some centres offer women a choice, although this might not be feasible for all centres.

Research and practice implications

Now that we have a record of current practice, more research is needed in order to fully understand what impact telephone results services have on women attending screening, and whether variations in the results giving process also have an impact. This would be particularly beneficial to consider for non-cancer results, where results are being routinely delivered by telephone to large numbers of women every year. Further research should also consider how women themselves might prefer to receive their results from screening and focus upon the patient perspective.

For peer review only

For peer review only - http://bmjopen.bmj.com/site/about/guidelines.xhtml

V.9 23.04.2019

Funding statement

This research is part of a PhD award and is funded by the Economic and Social Research Council (ESRC) Doctoral Training Centre at the University of Warwick. The funding has been awarded for this studentship to Sian Williamson for her PhD project for 4 years of full-time study. The award consists of payment of academic fees and a maintenance award.

A further contract between the University of Warwick, Public Health England (PHE) and the PhD Student (Sian Williamson) has secured £4,000 in research expenses.

Dr Taylor-Phillips is funded by an NIHR Career Development Fellowship. The views expressed in this paper are those of the authors and not the NIHR, PHE, the Department of Health and Social Care, or the Economic and Social Research Council (ESRC).

For peer review only

Contributor ship statement

SZW is the lead researcher. SZW, RJ, DE, STP and HS drafted the manuscript. SZW, RJ, HS, STP, JJ, MC and OK participated in the design of the study. SZW collected and analysed the data. OK and JJ assisted with the dissemination of the survey. All authors have reviewed and approved the manuscript.

Competing interests

None declared.

Data sharing statement

All data relevant to the study are included in the article.

Patient and public involvement

No patient involved.

For peer review only - http://bmjopen.bmj.com/site/about/guidelines.xhtml

V.9 23.04.2019

References

1. Ferlay J, Soerjomataram I, Dikshit R, Eser S, Mathers C, Rebelo M, et al. Cancer incidence and mortality worldwide: sources, methods and major patterns in GLOBOCAN 2012. 2015;136(5):E359-E86.
2. Keyzer-Dekker CM, De Vries J, van Esch L, Ernst MF, Nieuwenhuijzen GA, Roukema JA, et al. Anxiety after an abnormal screening mammogram is a serious problem. The Breast. 2012;21(1):83-8.
3. UK CR. Breast screening 2017 [cited 2018 5th March]. Available from: http://www.cancerresearchuk.org/about-cancer/breast-cancer/screening/breast-screening.
4. NHS Breast Screening Programme Central Return Data Set Overview (KC62). 2014-15.
5. Public Health England. Guidance - Clinical nurse specialists in breast screening. [Website]. 2019. [Available from: https://www.gov.uk/government/publications/breast-screening-guidance-for-clinical-nurse-specialists/clinical-nurse-specialists-in-breast-screening.
6. Public Health England. NHS public health functions agreement 2017- 18 Service specification no.24 Breast Screening Programme. 2017.
7. Chapman K. Interim quality assurance guidelines for clinical nurse specialists in breast cancer screening. 2012.
8. Andersen MR, Karlan BY, Drescher CW, Paley P, Hawley S, Palomares M, et al. False-positive screening events and worry influence decisions about surgery among high-risk women. 2019;38(1):43.
9. Choudhry A, Hong J, Chong K, Jiang B, Hartman R, Chu E, et al. Patients' preferences for biopsy result notification in an era of electronic messaging methods. Jama dermatology. 2015;151(5):513-21.
10. Liederman EM, Lee JC, Baquero VH, Seites PG. Patient-physician Web messaging. Journal of general internal medicine. 2005;20(1):52-7.
11. van Galen LS, Car JJb. Telephone consultations. 2018;360:k1047.
12. Currell R, Urquhart C, Wainwright P, Lewis R. Telemedicine versus face to face patient care: effects on professional practice and health care outcomes. Cochrane Database Syst Rev. 2000;2(2).
13. Williamson S, Patterson J, Crosby R, Johnson R, Sandhu H, Johnson S, et al. Communication of cancer screening results by letter, telephone or in person: A mixed methods systematic review of the effect on attendee anxiety, understanding and preferences. 2018.
14. Vaismoradi M, Turunen H, Bondas TJN, sciences h. Content analysis and thematic analysis: Implications for conducting a qualitative descriptive study. 2013;15(3):398-405.
15. Elo S, Kyngäs HJJoan. The qualitative content analysis process. 2008;62(1):107-15.
16. Cook SA, Salmon P, Hayes G, Byrne A, Fisher PLJPo. Predictors of emotional distress a year or more after diagnosis of cancer: A systematic review of the literature. 2018;27(3):791-801.
17. Seifart U, Baer T, Stumpenhorst M, Riera-Knorrenschild J, Seifart C. Breaking bad news-What patients want and what they get. Onkologie. 2011;34:46.
18. Appleby J, Harrison A, Devlin NJ. What is the real cost of more patient choice?: King's Fund London; 2003.

For peer review only - http://bmjopen.bmj.com/site/about/guidelines.xhtml

19. Heleno B, Siersma V, Brodersen J. Waiting time and the psychosocial consequences of false-positive mammography: cohort study. Journal of negative results in biomedicine. 2015;14(1):8.
20. Skotko BG, Kishnani PS, Capone GT, Group DSDS. Prenatal diagnosis of Down syndrome: How best to deliver the news. American journal of medical genetics. 2009;149(11):2361-7.

For peer review only

For peer review only - http://bmjopen.bmj.com/site/about/guidelines.xhtml

The communication of benign biopsy results in the NHS Breast Screening Programme – Online centre survey

CENTRE CHARACTERISTICS

1. Please state the official name of your breast screening centre:

   ____

2. What age group of women does your centre screen most frequently?
   (this information for your centre can be found in the KC62)

   ☐ <=44
   ☐ 45-49
   ☐ 50-52
   ☐ 53-54
   ☐ 55-59
   ☐ 60-64
   ☐ 65-69
   ☐ 70
   ☐ 71-74
   ☐ >=75

For peer review only

For peer review only - http://bmjopen.bmj.com/site/about/guidelines.xhtml

COMMUNICATION OF RESULTS

3. When delivering benign biopsy results…

   Women are never telephoned with results ☐
   Women are occasionally telephoned with results ☐
   Women are routinely telephoned with results ☐

   Please add any additional comments below:

____
____
____

4. If women ***are*** telephoned with benign results, who delivers these results? (Tick all that apply)

   Clinical nurse specialist ☐
   Radiologist ☐
   Radiographer ☐
   Breast care surgeon ☐
   Administrative staff ☐
   Other (please specify): ☐

   ____

1st April 2017 – V.3

For peer review only

For peer review only - http://bmjopen.bmj.com/site/about/guidelines.xhtml

5. When delivering a cancer result…

Women are never telephoned with results ☐
Women are occasionally telephoned with results ☐
Women are routinely telephoned with results ☐

Please add any additional comments below:

____
____
____

6. If women ***are*** telephoned with cancer results, who delivers these results? (Tick all that apply)

Clinical nurse specialist ☐
Radiologist ☐

Radiographer ☐
Breast care surgeon ☐

Administrative staff ☐

Other (please specify): ☐

____

1st April 2017 – V.3

For peer review only

For peer review only - http://bmjopen.bmj.com/site/about/guidelines.xhtml

7. From assessment to delivering a result, how long does this process take (on average)?
    - 1 day ☐
    - 2 days ☐
    - 3 days ☐
    - 4 days ☐
    - 5 days ☐
    - 6 days ☐
    - 7 days ☐
    - 8 days ☐
    - 9 days ☐
    - 10 days ☐
    - 11 days ☐
    - 12 days ☐
    - More than 12 days ☐

8. We are currently recruiting breast screening centres to take part in our main study, which will involve surveying women attending breast screening, who go to receive a benign result. The surveys will be assessing patient anxiety and understanding of results, examining whether the method of communication used (telephone or in-person) to deliver benign results influences these patient outcomes. We are looking for two centres to participate, to allocate women to receive benign results via either telephone or in-person. We are currently in the process of finalising the design of methodology and submitting for ethical approval.

    Would you be interested in involvement in this research?

    Yes ☐
    No ☐

If you select yes, you may be contacted soon with further details of the main study.

9. Would your centre like to know the results from this survey? (If yes, please provide an email address for a summary sheet to be sent upon completion of the research).

No ☐
Yes ☐

Email address: ___________________________________

1st April 2017 – V.3

For peer review only

Thank you for taking the time to participate in this survey.

If you have any questions, feel free to contact the lead researcher:

Lead researcher: Sian Williamson

Email: s.williamson@warwick.ac.uk

Telephone: 07891319509

For peer review only

1st April 2017 – V.3

Communicating biopsy results from breast screening assessment: Current practice in English breast screening centres and staff perspectives of telephoning results

Journal:	*BMJ Open*
Manuscript ID	bmjopen-2018-028683.R2
Article Type:	Original research
Date Submitted by the Author:	13-Aug-2019
Complete List of Authors:	Williamson, Sian; University of Warwick, Health Sciences<br>Johnson, Rebecca; Coventry University<br>Sandhu, Harbinder; University of Warwick Warwick Medical School, Clinical Trials Unit<br>Ellard, David; Warwick Medical School, Clinical trials Unit<br>Jenkins, Jacquie; Public Health England, National Programme Manager – NHS Breast Screening Programme<br>Casey, Margaret; Royal Wolverhampton NHS Trust, Clinical Nurse Specialist Breast Care<br>Kearins, Olive; Public Health England, National Lead Breast Screening QA<br>Taylor-Phillips, Sian; University of Warwick, Warwick Medical School
**Primary Subject Heading**:	Communication
Secondary Subject Heading:	Health policy, Health services research, Oncology, Public health
Keywords:	PREVENTIVE MEDICINE, Health policy < HEALTH SERVICES ADMINISTRATION & MANAGEMENT, QUALITATIVE RESEARCH, Quality in health care < HEALTH SERVICES ADMINISTRATION & MANAGEMENT

SCHOLARONE™
Manuscripts

V.9 23.04.2019

Back to top

Communicating biopsy results from breast screening assessment: Current practice in English breast screening centres and staff perspectives of telephoning results

Author list: Sian Williamson (PhD Student, University of Warwick), Rebecca Johnson (Senior Lecturer in Public Health, Coventry University), Harbinder Sandhu (Associate Professor, University of Warwick), David Ellard (Associate Professor, University of Warwick), Jacquie Jenkins (National Programme Manager – NHS Breast Screening Programme, Royal Wolverhampton NHS Trust), Margaret Casey (Clinical Nurse Specialist Breast Care), Olive Kearins (National Lead Breast Screening QA, Public Health England) & Sian Taylor-Phillips (Associate Professor, University of Warwick).

Corresponding author: Rebecca Johnson,
Coventry University
ad1658@coventry.ac.uk

Word count (excluding abstract and tables): 4,118 words

For peer review only - http://bmjopen.bmj.com/site/about/guidelines.xhtml

V.9 23.04.2019

Abstract (299 words)

Objective

To record how breast screening centres in England deliver all biopsy results (cancer/non-cancer) from the breast assessment visit.

Design

Online survey of 63 of 79 breast screening centres in England from all regions (East Midlands, East of England, London, North East Yorkshire & Humber, North West, South East, South West, West Midlands). The survey contained quantitative measures of frequency for telephoning biopsy results (routinely, occasionally or never) and optional qualitative free-text responses. Surveys were completed by a staff member from each centre.

Results

There were no regional trends in the use of telephone results services, (X^2 (14, N = 63) = 11.55, p =.64), Centres who telephoned results routinely did not deliver results sooner than centres who deliver results in-person (X^2 (16, N = 63) = 12.76, p =.69).

When delivering cancer results, 76.2% of centres never telephone results and 23.8% of centres occasionally telephone results. No centres reported delivering cancer results routinely by telephone. Qualitative content analysis suggest that cancer results are only telephoned at patient request and under exceptional circumstances.

When delivering non-cancer results, 12.7% of centres never telephoned results, 38.1% occasionally telephoned results and 49.2% routinely telephoned results. Qualitative content analysis revealed different processes for delivering telephone results, including patient choice and scheduling an in-person results appointment for all women attending breast assessment, then ringing non-cancer results unexpectedly ahead of this pre-booked appointment.

Conclusions

In the National Health Service Breast Screening Programme, breast assessment results that are cancer are routinely delivered in-person. However, non-cancer breast assessment results are often routinely delivered by telephone, despite breast screening policy recommendations.

More research is needed to understand the impact of telephoning results on women attending breast assessment, particularly women who receive a non-cancer result. Future research should also consider how women themselves might prefer to receive their results.

For peer review only - http://bmjopen.bmj.com/site/about/guidelines.xhtml

V.9 23.04.2019

Article summary

Strengths and limitations

- This study gives an up-to-date picture of current results-giving practice of National Health Service Breast Screening Programme centres nationally.
- A high response rate was achieved, so results are generalizable to the English National Health Service Breast Screening Programme.
- The qualitative comments made by National Health Service staff gave deeper insight into how telephone results are delivered in practice.
- Survey responses were subjective and not checked against centre policy documents.
- Formal analysis of the concordance between communication practice and policy guidelines was not conducted

For peer review only

For peer review only - http://bmjopen.bmj.com/site/about/guidelines.xhtml

V.9 23.04.2019

Communicating biopsy results from breast screening assessment: Current practice in English breast screening centres and staff perspectives of telephoning results

Background

Breast cancer is one of the most common cancers internationally (1). The National Health Service Breast Screening Programme (NHSBSP) was launched to aid the early detection of breast cancer at the population level, because early detection is linked with better prognosis (2). At screening, a mammogram (x-ray) is performed on each breast (3). If an abnormality is found during this screening mammogram, women will be recalled to attend assessment for further tests. These tests can include a core needle biopsy, which involves the removal of sections of tissue from the suspicious breast region which are sent for cytological examination. A biopsy is the definitive test at breast assessment to confirm if the mammogram abnormalities found are cancer. In 2016-17, 2,199,342 women in England were screened by the NHSBSP. 89.104 of these women were referred for assessment and 40, 255 women had a core biopsy (4).

In recent years, the NHSBSP has been considering which communication method might be preferable for delivering non-cancer biopsy results from breast assessment. The NHSBSP service specification recommends that all breast assessment results should be delivered in-person, which includes cancer and non-cancer results (5). Furthermore, the guidelines state that telephone results should only be used at the patients request and should not be standard practice (6). Despite these recommendations, some centres already routinely deliver non-cancer assessment results by telephone (5, 7). There is ongoing concern about the impact of delivering a non-cancer assessment result by telephone. One of the main concerns is how anxious women feel when receiving a result, even when the result is non-cancer (8). Another concern is the potential for miscommunication by telephone.

In other areas of healthcare, a variety of communication methods are used to deliver results, including in-person consultations, telephone, letters and email. Each method of communication used in results delivery presents different advantages, disadvantages and challenges in implementation. Results delivered in-person are often seen as the ‘gold standard’ (9). However, as technology advances, fewer

For peer review only

V.9 23.04.2019

healthcare results are now delivered in-person. Liederman et al. (10) stated that 'face-to-face contact is not necessary for effective communication' (pg. 52). Most commonly, results delivery is moving towards telephone and telemedicine (11). Despite this, Cochrane review evidence suggests that we still do not know enough about the impact of telephone results services on healthcare outcomes (12).

There is no current record of how breast centres in England deliver biopsy results from breast assessment. Despite policy recommendations, some centres appear to routinely deliver non-cancer results by telephone. Furthermore, it is assumed that cancer results are all delivered in-person as guidelines recommend. There is a currently lack of evidence about how often telephone results are used to deliver breast assessment results (13).

In this study, we aimed to record how breast screening centres in England deliver biopsy results from breast assessment and answer the following questions:

1) How often are telephone results delivered and by whom? Does this differ when results are non-cancer versus cancer?
2) Is there a time difference between results delivered by telephone versus results delivered in-person?

For peer review only

V.9 23.04.2019

Methods

Participants

A link to an online survey hosted by the Bristol Online Survey platform was sent to all breast screening centres in England on 2nd June 2017. At this time, 79 breast screening units existed in England, as confirmed by a list from the Quality Assurance Lead for Breast Screening. Data collection ended on 28th February 2018.

The survey link was distributed to the manager of each breast screening centre via the Quality Assurance lead for each region (East Midlands, East of England, London, North East Yorkshire & Humber, North West, South East, South West, West Midlands). The link was accompanied by a brief study description. The survey was completed by a representative member of staff from each breast screening centre.

Survey completion reminders were sent periodically by the Quality Assurance leads to nonresponding centres.

Ethical approval for the survey was obtained from the Biomedical & Scientific Research Ethics Committee at the University of Warwick (REGO-2017-1908).

Survey piloting and instrument

The survey was designed using a previous tool developed by Clinical Nurse Specialist in Breast Care (Margaret Casey) in combination with discussions with key stakeholders from the NHSBSP. Stakeholders included the programme manager for the NHSBSP, the Quality Assurance Lead for the NHSBSP, and a clinical nurse specialist in breast care. Following this, a draft version underwent three rounds of piloting: stakeholder review of content, stakeholder piloting of the online layout and cognitive interviewing with a layperson.

The main questions in the survey focused on recording how often biopsy results were telephoned. The first question asked the frequency of delivering benign (non-cancer) biopsy results by telephone (never/occasionally/routinely). The second question asked the frequency of delivering cancer biopsy results by telephone (never/occasionally/routinely). After these two questions, a free text box was added to allow responders to comment on the answers provided. The survey also recorded

For peer review only - http://bmjopen.bmj.com/site/about/guidelines.xhtml

V.9 23.04.2019

who is responsible within the team for delivering telephone results at the centre (Clinical Nurse Specialist, Radiologist, Radiographer, Breast Care Surgeon, Administrative Staff, Other).

One question recorded the amount of time taken between clinic assessment and the delivery of a result (options spanning between 1 day and more than 12 days). These data were collected to compare the length of time taken to deliver results for centres who delivered results by telephone routinely versus those who never deliver telephone results.

The survey included 9 questions. It was expected that the survey would take 10 minutes to complete (see Appendix 1 for full survey).

Data analysis

Quantitative

Data cleaning processes were implemented. This involved checking for missing data, coding centres by region and removing duplicate responses. Descriptive statistics and response rates were calculated.

Percentages for delivering non-cancer results and cancer results by telephone were calculated separately, alongside frequencies for who delivers each type of results.

To identify whether there were any regional trends in the delivery of telephone results, a chi-square was calculated, comparing region (East Midlands, East of England, London, North East Yorkshire & Humber, North West, South East, South West, West Midlands) with telephone frequency (routinely, occasionally, never).

To identify any potential time differences between telephone and in-person results a chi-square was calculated, comparing telephone frequency (routinely, occasionally, never) to length of time between assessment and results (1 day – 12+ days).

Statistical significance was assumed at $p<.05$. Statistical analysis was conducted using IBM SPSS Statistics 24 software.

For peer review only - http://bmjopen.bmj.com/site/about/guidelines.xhtml

V.9 23.04.2019

Qualitative

Qualitative free text comments were analysed using qualitative content analysis (14, 15). This approach allowed for commonalities amongst staff viewpoints to be identified and to be described narratively, in order to contextualise and expand upon the quantitative findings. Inter-coder reliability was used to ensure the rigour and trustworthiness of the analysis (14, 16). The analysis was conducted by the lead author (SW) and checked by a second author (DE) to ensure that the meaning of original staff comments was retained. Any disputes in interpretation were resolved by a third author (HS). Qualitative analysis was conducted using NVivo 12.

For peer review only

V.9 23.04.2019

Results

Respondents

Of the 79 breast screening centres in England, 63 (79%) responded to the online survey. All regions were represented in the quantitative survey (see Table 1).

Data relating to the mean age of women screened at each centre were removed from the data set due to 61.4% of responses being ‘I don’t know’.

For peer review only

V.9 23.04.2019

Table 1: Number of centres who responded to the online survey and provided optional qualitative comments

**Centres in each region (n=79)**[1]	**Centres who responded to the survey**[2] **(n=63)**	**Centres who commented on telephoning non-cancer results**[3] **(n=28)**	**Centres who commented on telephoning cancer results**[4] **(n=20)**
East Midlands (n=9)	7	5	3
East of England (n=11)	10	5	2
London (n=6)	4	0	0
North East Yorkshire & Humber (n=12)	10	3	1
North West (n=11)	8	2	2
South East (n=8)	4	2	3
South West (n=13)	11	5	6
West Midlands (n=9)	9	6	3

[1] The total number of centres within each region of England.
[2] The number of centres who completed the survey from each region, providing quantitative data.
[3] The number of centres who provided comments in the 1st qualitative free-text box, asking about telephoning non-cancer results. This was not a mandatory question and not all centres who completed the survey provided qualitative data.
[4] The number of centres who provided comments in the 2nd qualitative free-text box, asking about telephoning cancer results. This was not a mandatory question and not all centres who completed the survey provided qualitative data.

For peer review only - http://bmjopen.bmj.com/site/about/guidelines.xhtml

V.9 23.04.2019

Quantitative findings

Frequency of results by telephone

When delivering non-cancer results, the majority of centres routinely telephoned results (see Table 2) and most of these results were delivered by Clinical Nurse Specialists (see Table 3).

When delivering cancer results, the majority of centres never telephoned results (see Table 2). When cancer results were delivered by telephone, most of these results were delivered by Clinical Nurse Specialists (see Table 3).

Table 2: Frequency and percentage of centres who routinely, occasionally or never deliver results by telephone for non-cancer and cancer results (n=63)

	Routinely	Occasionally	Never
Non-cancer	31 (49.2%)	24 (38.1%)	8 (12.7%)
Cancer	0 (0%)	15 (23.8%)	48 (76.2%)

Table 3: Frequency of who delivers results by telephone, for non-cancer and cancer results (n=63)

	Clinical Nurse Specialist	Radiologist	Radiographer	Breast Surgeon	Administrative staff	Other
Non-cancer	42	17	5	5	0	2
Cancer	13	2	0	2	0	0

N.B. Centres could select multiple responses to this question.

For peer review only - http://bmjopen.bmj.com/site/about/guidelines.xhtml

Regional differences – frequency of telephone results

No relationship was found between region and the frequency of non-cancer telephone results, (X^2 (14, N = 63) = 11.55, p =.64). This indicates no regional trends in the use of telephone results services.

Time difference – telephone results vs. in-person results

The mean time to deliver results for all centres was 7.03 days (SD = 2.03). See Table 4 for all means (SDs). No relationship was found between frequency of telephone results and length of time from assessment to receipt of results, (X^2 (16, N = 63) = 12.76, p =.69). This indicates that centres delivering results by telephone do not deliver them sooner after the assessment visit than centres delivering results in-person (or never telephone).

Table 4: Mean (SDs) number of days between assessment and delivery of results for centres who routinely, occasionally and never benign (non-cancer) telephone results

	Routinely telephone	Occasionally telephone	Never telephone
Mean (SDs) number of days between assessment and delivery of results	6.77 (1.76)	7.13 (2.09)	7.75 (2.81)

For peer review only - http://bmjopen.bmj.com/site/about/guidelines.xhtml

V.9 23.04.2019

Qualitative findings

In the survey, NHS staff had the option to comment in free-text boxes after two questions. The results are presented in two sections, with one focusing on first question (non-cancer) and one focusing on the second question (cancer). All regions (excluding London) provided qualitative free-text responses (see Table 1). See Appendix 2 for a tabular representation of all qualitative comments from the content analysis.

For peer review only

When delivering benign (non-cancer) biopsy results are women never telephoned with results, occasionally telephoned with results or routinely telephoned with results?

This section presents the qualitative findings following the first survey question (n=28), which asked the frequency of delivering non-cancer biopsy results by telephone (never/occasionally/routinely).

Content analysis revealed that 7 centres scheduled for all women to return to receive results in-person. Then, if the test results for these women are confirmed as non-cancer, centres attempt to ring women with telephone results ahead of the pre-scheduled in-person appointment. Example comments include:

"Women have a scheduled face to face appointment for results but if it's benign we ring them." (Centre ID 02)

"Our aim is to call all the benign results and offer to cancel the booked appointment." (Centre ID 50)

"All women are given a results appointment during assessment clinic. Following MDT, all those with benign biopsy results are contacted by telephone. If contact is made, the result is discussed and the results appointment cancelled." (Centre ID 38)

1 centre (Centre ID 25) commented that they "normally see women face to face", with this being their routine practice. This suggests that breast screening centres differ in how they deliver non-cancer breast assessment results.

For peer review only - http://bmjopen.bmj.com/site/about/guidelines.xhtml

Option to still attend

Content analysis revealed 5 centres who commented that, when women are contacted by telephone with a non-cancer result, they are still offered the option to attend in-person if they have further questions. Example comments include:

“All patients are given an appointment to attend for results we do telephone with results but patients are still able to attend, and some do.” (Centre ID 45)

“After the MDT. Patients are telephoned with benign results by a qualified Breast Care Nurse. They are then offered an OPA with a consultant surgeon if they have concerns.” (Centre ID 31)

Rare and exceptional circumstances

Content analysis revealed 5 centres only deliver non-cancer results by telephone in exceptional circumstances. Example comments include:

“Only in exceptional circumstances” (Centre ID 59)

“This is not done routinely and very rarely occurs.” (Centre ID 78)

Reasons given for giving non-cancer results by telephone included if the woman finds it difficult to attend in-person and so the woman can be where she wants to be to receive results.

For peer review only

For peer review only - http://bmjopen.bmj.com/site/about/guidelines.xhtml

V.9 23.04.2019

Giving women a choice or at patient request

Content analysis revealed 5 centres ask women how they would like their result to be delivered if it is not cancer. Example comments include:

"We will always offer them an appointment to come in, but the BCNs will ask if they want a telephone call at the time of assessment" (Centre ID 63)

"Women are asked at assessment if they would like a telephone call or they can come back for results if they do not wished to be telephoned" (Centre ID 29)

"Women are given a choice about how they receive their results when the imaging suggests a benign process" (Centre ID 42)

One centre commented that non-cancer results are only delivered by telephone at the request of the patient:

"This is not routine practice but happens if a patient requests it and the probability of a benign result is very high." (Centre ID 73)

<u>Summary of non-cancer content analysis</u>

Content analysis revealed conflicting centre comments. Some centres schedule in-person results appointments for all women, but then attempt to contact women with non-cancer results by telephone instead. However, one centre commented that delivering non-cancer results by telephone was not routine practice. Other centres commented that telephoning non-cancer results only happens under exceptional circumstances, such as the woman being unable to attend in-person.

Content analysis revealed that some women who are telephoned with results are still offered the option to attend if they have further questions. Some centres ask women how they would prefer to be contacted with their result if it is not cancer.

For peer review only - http://bmjopen.bmj.com/site/about/guidelines.xhtml

V.9 23.04.2019

<u>When delivering cancer biopsy results are women never telephoned with results, occasionally telephoned with results or routinely telephoned with results?</u>

This section presents the qualitative findings following the second survey question n=20), which asked the frequency of delivering cancer biopsy results by telephone (never/occasionally/routinely).

Content analysis revealed 4 centres routinely deliver cancer results in-person. Example comments include:

"Cancer diagnoses are always communicated face to face." (Centre ID 39)

"[telephone results] This would never be planned." (Centre ID 09)

"All positive results or complicated cases are invited back to be given results by the Breast Surgery Team." (Centre ID 26)

Rare and exceptional circumstances

Content analysis revealed 11 centres only deliver cancer results by telephone in exceptional circumstances. Example comments include:

"Rarely telephoned with a cancer diagnosis always at the patients request in extenuating circumstances." (Centre ID 45)

"This is a rare occurrence and is only agreed to with the patients prior consent on the understanding they may be receiving a cancer diagnosis." (Centre ID 11).

"Very rare - this would only happen with prior agreement if a woman is to be away for an extended period of time." (Centre ID 38)

Reasons for delivering cancer results by telephone under these exceptional circumstances were if the woman finds it difficult to attend in-person and if the woman was going to be away for an extended period of time.

For peer review only - http://bmjopen.bmj.com/site/about/guidelines.xhtml

V.9 23.04.2019

Giving women a choice or at patient request

Content analysis revealed 2 centres ask women how they would like their result to be delivered if it is cancer. Example comments include:

"They are asked if the result was a surprise and was a breast cancer would you still wish to get that news over the phone." (Centre ID 01)

"Women are asked at assessment if they would like a telephone call or they can come back for results if they do not wished to be telephoned. (Centre ID 29)"

Content analysis revealed 5 centres only deliver cancer results by telephone at the request of the patient. Example comments include:

***"**Patient request only" (Centre ID 33)*

"Only very rarely and at patient's specific request" (Centre ID 08)

Unexpected results and a negative reaction

One centre (Centre ID 60) addressed the issue of how to deal with an unexpected result. At the breast assessment visit, this centre informs women with a high suspicion of a non-cancer result that they will have their result delivered by telephone. However, if *"If there is a positive result which was unexpected a Breast Care Nurse rings the woman to advise an appointment is required to discuss the results".*

One centre (Centre ID 15) commented about a woman who *"reacted extremely badly on telephone"* when receiving a cancer result.

For peer review only - http://bmjopen.bmj.com/site/about/guidelines.xhtml

V.9 23.04.2019

Summary of cancer content analysis

For cancer results, in-person results are routine. Telephoning cancer results is only being offered under rare or exceptional circumstances such as when women have difficulty in attending. These telephone results are only given at the request of the patient.

Content analysis revealed the potential difficulties in delivering and scheduling results by telephone. One comment highlighted the negative reaction of a woman who received her cancer result by telephone. Another comment addressed the issue of how to deal with a result which was presumed to be non-cancer but turned out to be cancer.

For peer review only

For peer review only - http://bmjopen.bmj.com/site/about/guidelines.xhtml

V.9 23.04.2019

Discussion

The aim of this research was to record how breast screening centres in England deliver biopsy results from breast assessment, by assessing how often telephone results are delivered and by whom. This research also aimed to see if telephone results delivery differs when assessment results are non-cancer versus cancer. Furthermore, this research aimed to assess if there is a time difference between results delivered by telephone versus results delivered in-person.

Our research suggests that centres routinely delivering results by telephone do not deliver them sooner than centres who deliver results in-person. This contradicts articles citing 'speed' as one of the potential advantages of telephone results (9, 17, 18).

Our study found that delivering cancer results by telephone is not common practice for breast screening centres in England. Telephoning cancer results is only used in exceptional circumstances and only at the request of the patient. For example, telephone results might be used if the woman is physically unable to attend in-person (e.g. health issues or away from the country for an extended period). When cancer results are telephoned, most of the results are delivered by Clinical Nurse Specialists. The reason why cancer results are rarely telephoned is probably due to the emotional impact of receiving a cancer diagnosis (19). The extensive literature on "breaking bad news" in healthcare places importance on the location where results are received to ensure no disturbances (20). This may help to explain the one comment in the current study where a centre reported the negative reaction of a woman who received a cancer result by telephone. However, this comment was only made by one centre in the study and may not be representative of the population as a whole so this finding should be interpreted with caution.

Another comment in the study highlighted the issues of how to deal with a result which was presumed to be non-cancer at the assessment stage but turned out to be cancer. At the stage of breast assessment, it is unknown if a woman will receive a cancer or non-cancer result. However, breast assessment tests may indicate a higher chance of a non-cancer result. These women might be informed of the lower likelihood of cancer and are offered the opportunity to receive results by telephone. If the biopsy result then confirms an unexpected cancer, women may then be

For peer review only - http://bmjopen.bmj.com/site/about/guidelines.xhtml

telephoned with a cancer result. This may have implications for anxiety and may be avoided by not offering telephone results. However, this was an issue only highlighted by one centre in the study and does not represent the centre comments as a whole.

Our study found that delivering non-cancer results by telephone is routine practice for roughly half of the breast screening centres in England, with most of the results being delivered by Clinical Nurse Specialists. This appears to contrast with breast screening policy guidelines, which state that telephone results should only be used at the patients request and should not be routinely offered. However, the qualitative findings clarified that some of these centres offer women a choice of how they would prefer to receive their results. This suggests that some centres are still acting within the guidelines by only telephoning women who choose this communication. Offering women the choice between telephone and in-person communication may not be feasible for all centres (21). For centres who already routinely telephone results, there may be a reduced capacity to provide results in-person if this is requested by the woman. From the content analysis in the current study, a compromise is to telephone all non-cancer results routinely but to also offer the option for the woman to still attend the clinic in-person. Offering patients a choice of communication method of results at the assessment visit might also be problematic due to heightened anxiety with some women not wanting to make a decision (22, 23).

Some centres who routinely deliver non-cancer results by telephone do not offer patient choice. A common practice is for centres to give all women who attend breast assessment an in-person appointment to return for results, but then telephone women with non-cancer results ahead of this scheduled appointment. The centres commented that this process has the potential to reduce the expected wait time for women to receive results, thus minimising the amount of time spent anxiously waiting (24). However, the psychological impact of receiving an ‘unexpected’ communication method has not been considered in this setting. When a telephone result is not expected, it is possible that women may feel unprepared or not in the right mind-set to comprehend the information given (25). This may contribute to the anxiety associated with screening and might be an avoidable harm. However, from the current research we do not know if this is the case.

For peer review only - http://bmjopen.bmj.com/site/about/guidelines.xhtml

V.9 23.04.2019

Strengths

This study formally reports the national communication practice of NHSBSP centres for delivering non-cancer and cancer breast assessment results. The high response rate indicated that this is an important issue for staff working within the screening programme.

The content analysis of qualitative comments allowed for expansion of quantitative survey data, which gave greater insight into how telephone results were implemented in practice.

Limitations

The time difference between telephone results and in-person results was quantified to allow the survey to be easily answered. This was quantified by the difference in days between clinic assessment and the delivery of a result for centres who either routinely, occasionally or never telephone results. However, other factors could be involved in the speed of results delivery.

The survey responses completed by centre staff were subjective and could not be validated by records. Furthermore, the staff member completing each survey was not recorded as part of the study. Therefore, quantitative data may not accurately reflect current communication practice due to the potential for human error or level of experience of the staff member completing the survey.

It is possible that centres who did not respond to the survey may be systematically different from centres who did. Therefore, the results may not be representative of all breast screening centres in England. However, the high response rate limits this issue.

A formal analysis of the concordance between communication practice and policy guidelines was not conducted. This was considered in the discussion but future research could consider a formal comparison-based analysis.

For peer review only - http://bmjopen.bmj.com/site/about/guidelines.xhtml

V.9 23.04.2019

Conclusion

In the NHSBSP, breast assessment results that are cancer are routinely delivered in-person, as recommended by policy guidelines. However, non-cancer breast assessment results are often routinely delivered by telephone despite the recommendations made in policy guidelines. Despite this, telephone results do not appear to be quicker than in-person results in practice.

A common process is to give all women attending breast assessment an appointment to come back to receive results in-person, to then telephone all women with non-cancer results ahead of this appointment. Some centres offer women a choice, although this might not be feasible for all centres and it is possible that women might be too anxious to make an informed decision.

Research and practice implications

Now that we have a record of current practice, more research is needed in order to fully understand what impact telephone results services have on women attending breast assessment, and whether variations in the results giving process also have an impact. This would be particularly beneficial to consider for non-cancer results, where results are being routinely delivered by telephone to large numbers of women every year. Further research should also consider how women themselves might prefer to receive their results and focus upon the patient perspective.

For peer review only - http://bmjopen.bmj.com/site/about/guidelines.xhtml

V.9 23.04.2019

Funding statement

This research is part of a PhD award and is funded by the Economic and Social Research Council (ESRC) Doctoral Training Centre at the University of Warwick. The funding has been awarded for this studentship to Sian Williamson for her PhD project for 4 years of full-time study. The award consists of payment of academic fees and a maintenance award.

A further contract between the University of Warwick, Public Health England (PHE) and the PhD Student (Sian Williamson) has secured £4,000 in research expenses.

Dr Taylor-Phillips is funded by an NIHR Career Development Fellowship. The views expressed in this paper are those of the authors and not the NIHR, PHE, the Department of Health and Social Care, or the Economic and Social Research Council (ESRC).

For peer review only

Contributor ship statement

SZW is the lead researcher. SZW, RJ, DE, STP and HS drafted the manuscript. SZW, RJ, HS, STP, JJ, MC and OK participated in the design of the study. SZW collected and analysed the data. OK and JJ assisted with the dissemination of the survey. All authors have reviewed and approved the manuscript.

Competing interests

None declared.

Data sharing statement

All data relevant to the study are included in the article.

Patient and public involvement

No patient involved.

For peer review only - http://bmjopen.bmj.com/site/about/guidelines.xhtml

V.9 23.04.2019

References

1. Ferlay J, Soerjomataram I, Dikshit R, Eser S, Mathers C, Rebelo M, et al. Cancer incidence and mortality worldwide: sources, methods and major patterns in GLOBOCAN 2012. International journal of cancer. 2015;136(5):E359-E86.
2. Keyzer-Dekker CM, De Vries J, van Esch L, Ernst MF, Nieuwenhuijzen GA, Roukema JA, et al. Anxiety after an abnormal screening mammogram is a serious problem. The Breast. 2012;21(1):83-8.
3. M. M. Understanding benign breast disorders and disease. Nursing Standard. 2001;15(50):45-52.
4. NHS Breast Screening Programme Central Return Data Set Overview (KC62). 2016-17.
5. England. PH. Guidance - Clinical nurse specialists in breast screening. [Website]. 2019. [Available from: https://www.gov.uk/government/publications/breast-screening-guidance-for-clinical-nurse-specialists/clinical-nurse-specialists-in-breast-screening.
6. Public Health England. NHS public health functions agreement 2017- 18 Service specification no.24 Breast Screening Programme. In: England. PH, editor. 2017.
7. Chapman K. Interim quality assurance guidelines for clinical nurse specialists in breast cancer screening. 2012.
8. Andersen MR, Karlan BY, Drescher CW, Paley P, Hawley S, Palomares M, et al. False-positive screening events and worry influence decisions about surgery among high-risk women. Health psychology. 2019;38(1):43.
9. Choudhry A, Hong J, Chong K, Jiang B, Hartman R, Chu E, et al. Patients' preferences for biopsy result notification in an era of electronic messaging methods. JAMA dermatology. 2015;151(5):513-21.
10. Liederman EM, Lee JC, Baquero VH, Seites PG. Patient-physician Web messaging. Journal of general internal medicine. 2005;20(1):52-7.
11. van Galen LS, Car J. Telephone consultations. BMJ. 2018;360:k1047.
12. Currell R, Urquhart C, Wainwright P, Lewis R. Telemedicine versus face to face patient care: effects on professional practice and health care outcomes. Cochrane Database of Systematic Reviews. 2000;2(2).
13. Williamson S, Patterson J, Crosby R, Johnson R, Sandhu H, Johnson S, et al. Communication of cancer screening results by letter, telephone or in person: A mixed methods systematic review of the effect on attendee anxiety, understanding and preferences. 2018.
14. Vaismoradi M, Turunen H, Bondas T. Content analysis and thematic analysis: Implications for conducting a qualitative descriptive study. Nursing Health Sciences. 2013;15(3):398-405.
15. Elo S, Kyngäs H. The qualitative content analysis process. Journal of advanced nursing. 2008;62(1):107-15.
16. Cavanagh S. Content analysis: concepts, methods and applications. Nurse researcher. 1997;4(3):5-16.
17. Purnell CA, Arnold RM. Retrospective analysis of communication with patients undergoing radiological breast biopsy. The journal of supportive oncology. 2010;8(6):259-63.
18. Leekha S, Thomas KG, Chaudhry R, Thomas MR. Patient preferences for and satisfaction with methods of communicating test results in a primary care

For peer review only

V.9 23.04.2019

practice. The Joint Commission Journal on Quality and Patient Safety. 2009;35(10):497-AP4.
19. Cook SA, Salmon P, Hayes G, Byrne A, Fisher PL. Predictors of emotional distress a year or more after diagnosis of cancer: A systematic review of the literature. Psycho-oncology. 2018;27(3):791-801.
20. Seifart U, Baer T, Stumpenhorst M, Riera-Knorrenschild J, Seifart C. Breaking bad news-What patients want and what they get. Onkologie. 2011;34:46.
21. Appleby J, Harrison A, Devlin NJ. What is the real cost of more patient choice?: King's Fund London; 2003.
22. Levinson W, Kao A, Kuby A, Thisted RA. Not all patients want to participate in decision making: a national study of public preferences. Journal of general internal medicine. 2005;20(6):531-5.
23. Brock DW, Wartman SA. When competent patients make irrational choices. Mass Medical Soc; 1990.
24. Heleno B, Siersma V, Brodersen J. Waiting time and the psychosocial consequences of false-positive mammography: cohort study. Journal of negative results in biomedicine. 2015;14(1):8.
25. Skotko BG, Kishnani PS, Capone GT, Group DSDS. Prenatal diagnosis of Down syndrome: How best to deliver the news. American journal of medical genetics. 2009;149(11):2361-7.

For peer review only

For peer review only - http://bmjopen.bmj.com/site/about/guidelines.xhtml

The communication of benign biopsy results in the NHS Breast Screening Programme – Online centre survey

CENTRE CHARACTERISTICS

1. Please state the official name of your breast screening centre:

   ____

2. What age group of women does your centre screen most frequently?
   (this information for your centre can be found in the KC62)

   ☐ <=44

   ☐ 45-49

   ☐ 50-52

   ☐ 53-54

   ☐ 55-59

   ☐ 60-64

   ☐ 65-69

   ☐ 70

   ☐ 71-74

   ☐ >=75

For peer review only

For peer review only - http://bmjopen.bmj.com/site/about/guidelines.xhtml

COMMUNICATION OF RESULTS

3. When delivering benign biopsy results…

   - Women are never telephoned with results ☐
   - Women are occasionally telephoned with results ☐
   - Women are routinely telephoned with results ☐

   Please add any additional comments below:

   ____
   ____
   ____

4. If women ***are*** telephoned with benign results, who delivers these results? (Tick all that apply)

   - Clinical nurse specialist ☐
   - Radiologist ☐
   - Radiographer ☐
   - Breast care surgeon ☐
   - Administrative staff ☐
   - Other (please specify): ☐

   ____

1st April 2017 – V.3

For peer review only

5. When delivering a cancer result…

Women are never telephoned with results ☐
Women are occasionally telephoned with results ☐
Women are routinely telephoned with results ☐

Please add any additional comments below:

____
____
____

6. If women ***are*** telephoned with cancer results, who delivers these results? (Tick all that apply)

Clinical nurse specialist ☐
Radiologist ☐

Radiographer ☐
Breast care surgeon ☐

Administrative staff ☐

Other (please specify): ☐

____

1st April 2017 – V.3

For peer review only - http://bmjopen.bmj.com/site/about/guidelines.xhtml

7. From assessment to delivering a result, how long does this process take (on average)?
   - 1 day ☐
   - 2 days ☐
   - 3 days ☐
   - 4 days ☐
   - 5 days ☐
   - 6 days ☐
   - 7 days ☐
   - 8 days ☐
   - 9 days ☐
   - 10 days ☐
   - 11 days ☐
   - 12 days ☐
   - More than 12 days ☐

8. We are currently recruiting breast screening centres to take part in our main study, which will involve surveying women attending breast screening, who go to receive a benign result. The surveys will be assessing patient anxiety and understanding of results, examining whether the method of communication used (telephone or in-person) to deliver benign results influences these patient outcomes. We are looking for two centres to participate, to allocate women to receive benign results via either telephone or in-person. We are currently in the process of finalising the design of methodology and submitting for ethical approval.

   Would you be interested in involvement in this research?

   - Yes ☐
   - No ☐

If you select yes, you may be contacted soon with further details of the main study.

9. Would your centre like to know the results from this survey? (If yes, please provide an email address for a summary sheet to be sent upon completion of the research).

No ☐
Yes ☐

Email address: ______________________________

1st April 2017 – V.3

For peer review only

Thank you for taking the time to participate in this survey.

If you have any questions, feel free to contact the lead researcher:

Lead researcher: Sian Williamson

Email: s.williamson@warwick.ac.uk

Telephone: 07891319509

For peer review only

For peer review only - http://bmjopen.bmj.com/site/about/guidelines.xhtml

Appendix 2 – Qualitative content analysis data in tabular form

When delivering benign (non-cancer) biopsy results are women never telephoned with results, occasionally telephoned with results or routinely telephoned with results?

Content	Comment
Centres schedule an in-person appointment for the woman to receive results but then telephone ahead of this appointment if results are benign (n=7)	*"Women have a scheduled face to face appointment for results but if it's benign we ring them." (Centre ID 02)*<br><br>*"Our aim is to call all the benign results and offer to cancel the booked appointment." (Centre ID 50)*<br><br>*"All women are given a results appointment during assessment clinic. Following MDT, all those with benign biopsy results are contacted by telephone. If contact is made, the result is discussed and the results appointment cancelled." (Centre ID 38)*<br><br>*"Women are seen in the assessment clinic and are asked by the BCN for permission to ring if need be to alter appointments etc. but we do not inform them that we will with the results in case it is more appropriate for them to come to clinic to receive the results, all patients leave clinic with an appointment" (Centre ID 34)*<br><br>*"All patients are given an appointment to attend for results we do telephone with results." (Centre ID 45)*<br><br>*"Women always have an appointment booked [but are telephoned if benign]" – (Centre ID 28)*<br><br>*"Appointments are made for all results but benign results are sometimes delivered over the phone, where women have been expecting a benign result." – (Centre ID 09)*
In-person communication is routine practice for delivering benign results (n=1)	*"We normally see women face to face." (Centre ID 25)*
Centres who telephone with results offer the woman the option to still attend in-person for further support (n=5)	*"All patients are given an appointment to attend for results we do telephone with results but patients are still able to attend, and some do." (Centre ID 45)*<br><br>*"After the MDT. Patients are telephoned with benign results by a qualified Breast Care Nurse. They are then offered an OPA with a consultant surgeon if they have concerns." (Centre ID 31)*<br><br>*"Our aim is to call all the benign results and offer to cancel the booked appointment, but we always stress they can come in anyway –no-one ever does!" (Centre ID 50)*<br><br>*"Women always have an appointment booked and are still offered to come to see a clinician if they still wish." (Centre ID 28)*<br><br>*"All women are given a results appointment during assessment clinic. Following MDT, all those with benign biopsy results are contacted by telephone. If contact is made, the result is discussed and the results appointment cancelled unless the woman specifically wants to attend for further information/support." (Centre ID 38)*
Centres only telephone benign results in rare and exceptional circumstances, where the woman cannot return in-person (n=5)	*"Only in exceptional circumstances." (Centre ID 59)*<br><br>*"This is not done routinely and very rarely occurs." (Centre ID 78)*<br><br>*"In exceptional circumstances." (Centre ID 46)*<br><br>*"Majority return for results, only rung if they have issues returning." (Centre ID 24)*<br><br>*"We normally see ladies face to face but will phone them if they are unable to attend their appointment." (Centre ID 25)*
Centres offer women the choice	*"We will always offer them an appointment to come in, but the BCNs will ask if they want a telephone call at the time of assessment." (Centre ID 63)*

For peer review only

of how they would prefer to receive their result (n=5)	*"Women are asked at assessment if they would like a telephone call or they can come back for results if they do not wished to be telephoned." (Centre ID 29)*<br><br>*"Women are given a choice about how they receive their results when the imaging suggests a benign process." (Centre ID 42)*<br><br>*"Women call in to us for a result as prearranged with specialist nurses. So they are where they want to be to get the result. The BCN will discuss with them beforehand the different outcomes that are possible. It is always explained that the result maybe different than expected and how they would feel about that." (Centre ID 01)*<br><br>*Prior discussion with the patient – (Centre ID 46)*
Centres will telephone women if the patient makes this request (n=1)	*"This is not routine practice but happens if a patient requests it and the probability of a benign result is very high." (Centre ID 73)*

For peer review only

When delivering cancer biopsy results are women never telephoned with results, occasionally telephoned with results or routinely telephoned with results?

Content	Comment
In-person communication is routine practice for delivering cancer results (n=4)	*"Cancer diagnoses are always communicated face to face." (Centre ID 39)*<br><br>*"[telephone results] This would never be planned." (Centre ID 09)*<br><br>*"All positive results or complicated cases are invited back to be given results by the Breast Surgery Team." (Centre ID 26)*<br><br>*"Routinely they are brought back for a face to face appointment." (Centre ID 02)*
Centres only telephone cancer results in rare and exceptional circumstances, where the woman cannot return in-person (n=11)	*"Rarely telephoned with a cancer diagnosis always at the patients request in extenuating circumstances." (Centre ID 45)*<br><br>*"This is a rare occurrence and is only agreed to with the patients prior consent on the understanding they may be receiving a cancer diagnosis." (Centre ID 11)*<br><br>*"Very rare - this would only happen with prior agreement if a woman is to be away for an extended period of time." (Centre ID 38)*<br><br>*"On rare occasions." (Centre ID 05)*<br><br>*"Except on one occasion." (Centre ID 15)*<br><br>*"Cancer results only discussed by telephone in the event that a woman refuses a face to face clinic appointment." (Centre ID 76)*<br><br>*"In exceptional circumstances." (Centre ID 46)*<br><br>*"Only in extreme circumstances and with much counselling beforehand - such as will be on holiday for months. This happens maybe once or twice a year." (Centre ID 50)*<br><br>*"Only very occasionally in exceptional circumstances." (Centre ID 17)*<br><br>*"This is very rare." (Centre ID 18)*<br><br>*"Only very rarely." (Centre ID 08)*
Centres offer women the choice of how they would prefer to receive their result (n=2)	*"They are asked if the result was a surprise and was a breast cancer would you still wish to get that news over the phone." (Centre ID 01)*<br><br>*"Women are asked at assessment if they would like a telephone call or they can come back for results if they do not wished to be telephoned." (Centre ID 29)*
Centres will telephone women if the patient makes this request (n=5)	*"Patient request only." (Centre ID 33)*<br><br>*"At patient's specific request." (Centre ID 08)*<br><br>*"At patient request." (Centre ID 05)*<br><br>*"Women are only telephoned with a cancer result if they have specifically asked for it that way and signed to give their consent." (Centre ID 02)*<br><br>*"Always at the patients request in extenuating circumstances." (Centre ID 45)*
What happens when a benign result is expected, a telephone appointment is arranged but the outcome is an unexpected cancer (n=1)	*"If there is a positive result which was unexpected a Breast Care Nurse rings the woman to advise an appointment is required to discuss the results." (Centre ID 60)*

For peer review only

A negative patient reaction to receiving cancer diagnosis by telephone (n=1)	*"Reacted extremely badly on telephone" (Centre ID 15)*

For peer review only
